# Transcriptional regulatory control of mammalian nephron progenitors revealed by multi-factor cistromic analysis and genetic studies

**DOI:** 10.1371/journal.pgen.1007181

**Published:** 2018-01-29

**Authors:** Lori L. O’Brien, Qiuyu Guo, Emad Bahrami-Samani, Joo-Seop Park, Sean M. Hasso, Young-Jin Lee, Alan Fang, Albert D. Kim, Jinjin Guo, Trudy M. Hong, Kevin A. Peterson, Scott Lozanoff, Ramya Raviram, Bing Ren, Ben Fogelgren, Andrew D. Smith, Anton Valouev, Andrew P. McMahon

**Affiliations:** 1 Department of Stem Cell Biology and Regenerative Medicine, Broad-CIRM Center, Keck School of Medicine, University of Southern California, Los Angeles, California, United States of America; 2 Department of Preventative Medicine, Division of Bioinformatics, Keck School of Medicine, University of Southern California, Los Angeles, California, United States of America; 3 Department of Molecular and Computational Biology, University of Southern California, Los Angeles, California, United States of America; 4 Division of Pediatric Urology and Division of Developmental Biology, Cincinnati Children's Hospital Medical Center, Cincinnati, Ohio, United States of America; 5 Department of Anatomy, Biochemistry, and Physiology, University of Hawaii at Manoa, Honolulu, Hawaii, United States of America; 6 The Jackson Laboratory, Bar Harbor, Maine, United States of America; 7 Ludwig Institute for Cancer Research, Department of Cellular and Molecular Medicine, Institute of Genomic Medicine, Moores Cancer Center, University of California San Diego La Jolla, California, United States of America; Seattle Children's Research Institute, UNITED STATES

## Abstract

Nephron progenitor number determines nephron endowment; a reduced nephron count is linked to the onset of kidney disease. Several transcriptional regulators including Six2, Wt1, Osr1, Sall1, Eya1, Pax2, and Hox11 paralogues are required for specification and/or maintenance of nephron progenitors. However, little is known about the regulatory intersection of these players. Here, we have mapped nephron progenitor-specific transcriptional networks of Six2, Hoxd11, Osr1, and Wt1. We identified 373 multi-factor associated ‘regulatory hotspots’ around genes closely associated with progenitor programs. To examine their functional significance, we deleted ‘hotspot’ enhancer elements for *Six2* and *Wnt4*. Removal of the distal enhancer for *Six2* leads to a ~40% reduction in *Six2* expression. When combined with a *Six2* null allele, progeny display a premature depletion of nephron progenitors. Loss of the *Wnt4* enhancer led to a significant reduction of *Wnt4* expression in renal vesicles and a mildly hypoplastic kidney, a phenotype also enhanced in combination with a *Wnt4* null mutation. To explore the regulatory landscape that supports proper target gene expression, we performed CTCF ChIP-seq to identify insulator-boundary regions. One such putative boundary lies between the *Six2* and *Six3* loci. Evidence for the functional significance of this boundary was obtained by deep sequencing of the radiation-induced *Brachyrrhine* (*Br*) mutant allele. We identified an inversion of the *Six2/Six3* locus around the CTCF-bound boundary, removing *Six2* from its distal enhancer regulation, but placed next to *Six3* enhancer elements which support ectopic *Six2* expression in the lens where *Six3* is normally expressed. *Six3* is now predicted to fall under control of the *Six2* distal enhancer. Consistent with this view, we observed ectopic Six3 in nephron progenitors. 4C-seq supports the model for *Six2* distal enhancer interactions in wild-type and *Br/+* mouse kidneys. Together, these data expand our view of the regulatory genome and regulatory landscape underpinning mammalian nephrogenesis.

## Author summary

Nephrons, the filtering units of the kidney, derive from nephron progenitors. Deficiencies in nephron number increases the risk of kidney disease. An understanding of the regulatory programs governing progenitor actions has important translational potential. Several transcription factors regulate the nephron progenitor population. However, their target interactions are largely unknown. Here, we mapped and intersected the genome-wide binding sites for four such factors in mouse nephron progenitor cells in the developing kidney: Six2, Hoxd11, Osr1, and Wt1. The intersectional data highlight a high-value set of putative enhancer elements linked to genes regulating nephron progenitor properties. We validate the function of two such enhancer elements regulating the levels of *Six2*, a key transcriptional regulatory factor in nephron progenitor maintenance, and *Wnt4*, a critical signaling factor controlling the mesenchyme to epithelial transition of induced nephron progenitors. Further characterization of the *Six2* regulatory landscape identified higher order regulatory interactions that ensure appropriate enhancer-promoter specificity. CTCF-bound sites between *Six2* and the adjacent *Six3* locus likely act as boundary elements to define topological interactions domains separating enhancer elements thereby providing distinct tissue specificity to each gene’s expression. An inversion of this region in the *Brachyrrhine* (*Br*) mutant mouse reverses *Six2* and *Six3* expression domains, placing *Six3* under control of the *Six2* enhancer element above resulting in kidney-specific expression, while *Six2* expression shifts to the lens, a normal expression domain for *Six3*. Together, these data expand our view of the regulatory genome and regulatory landscape underpinning mammalian nephrogenesis.

## Introduction

The mammalian metanephric kidney maintains fluid homeostasis. The number of individuals afflicted with kidney disease is on the rise, and reduced nephron number has been associated with disease outcome [1]. In the mouse, genetic studies have demonstrated that nephrons are generated from a Six2+ progenitor pool in a regulatory process requiring the transcriptional action of Six2 for progenitor maintenance [2]. Human SIX2 shows an expression and activity similar to its murine counterpart suggesting that mouse Six2 and human SIX2 likely have similar functions [3]. Consistent with this view, human mutations in *SIX2* are associated with renal hypodysplasia and the malignant transformation of progenitor cells in Wilms’ tumor, a pediatric nephroblastoma [4–6]. There is an increasing interest in the relationship between nephron progenitors, their output, and congenital and acquired kidney disease [1, 7]. Further, new approaches to modulate nephron progenitor outputs to generate kidney structures *in vitro* call for a better understanding of regulatory processes at play *in vivo* [8–10]. Nephron progenitor specification and nephron progenitor maintenance are dependent on a number of additional transcriptional regulatory factors including Hoxa/c/d11, Osr1, Wt1, Sall1, Eya1, Pax2, and Six1. Previous studies of mouse mutants in these genes suggest complex hierarchical interactions amongst these factors [11–27]. Identification of their genomic targets and target regulatory mechanisms are essential to determine the nephrogenic regulatory network.

Direct nephron progenitor ChIP-seq studies have identified a broad range of potential transcriptional targets of Six2/SIX2 action in the mouse and human kidney, respectively, and verified predicted enhancer modules for several of these targets [3, 28, 29]. Six2 interacts at cis-regulatory modules of genes expressed both in the nephron progenitors and their committed nephron-forming descendants through enhancers co-engaged by differentiation-inducing transcriptional complexes formed in response to canonical Wnt signaling [28, 29]. Interestingly, a potential role for Hox11 paralogs within Six2-predicted cis-regulatory modules is suggested by the strong enrichment of AT-rich homeobox motifs in Six2 ChIP-seq peaks [28]. The genomic targets of Wt1 have also been analyzed by ChIP experiments of embryonic mouse kidneys [30–32]. Though the approach was not specific to nephron progenitors, these studies revealed the interplay with many genes expressed in, and critical for, nephron progenitors, including Fgf and Bmp family members [30–32]. Sall1 ChIP-seq has also shed light on its active roles in nephron progenitors and repressive actions on development of nascent nephrons, respectively [29]. Interestingly, a subset of Six2- and Sall1-bound regions overlap suggesting these factors co-associate and target analysis predicts genes regulating the nephron progenitor population [29].

With a working model that multi-factor binding will highlight key regulatory nodes of the nephron progenitor pathway [33–37], we utilized ChIP-seq analysis to identify a subset of putative regulatory elements associated with multiple transcription factors. gRNA/Cas9-mediated ablation of ‘regulatory hotspots’ adjacent to *Six2* and *Wnt4* highlight the significance of these enhancer elements in regulating target gene expression. Additional analyses of the regulatory landscape surrounding *Six2* identified insulator-bound elements which constrain enhancer function. In support of this finding, deep sequencing of the *Br* mutant mouse identified an inversion of *Six2* and *Six3* loci altering enhancer specificity. These studies highlight the critical role of multi-factor input and proper enhancer context for directing appropriate target gene expression.

## Results

### Identification of nephron progenitor-specific transcription factor interaction sites using a novel transgenic strategy

**Fig 1:**
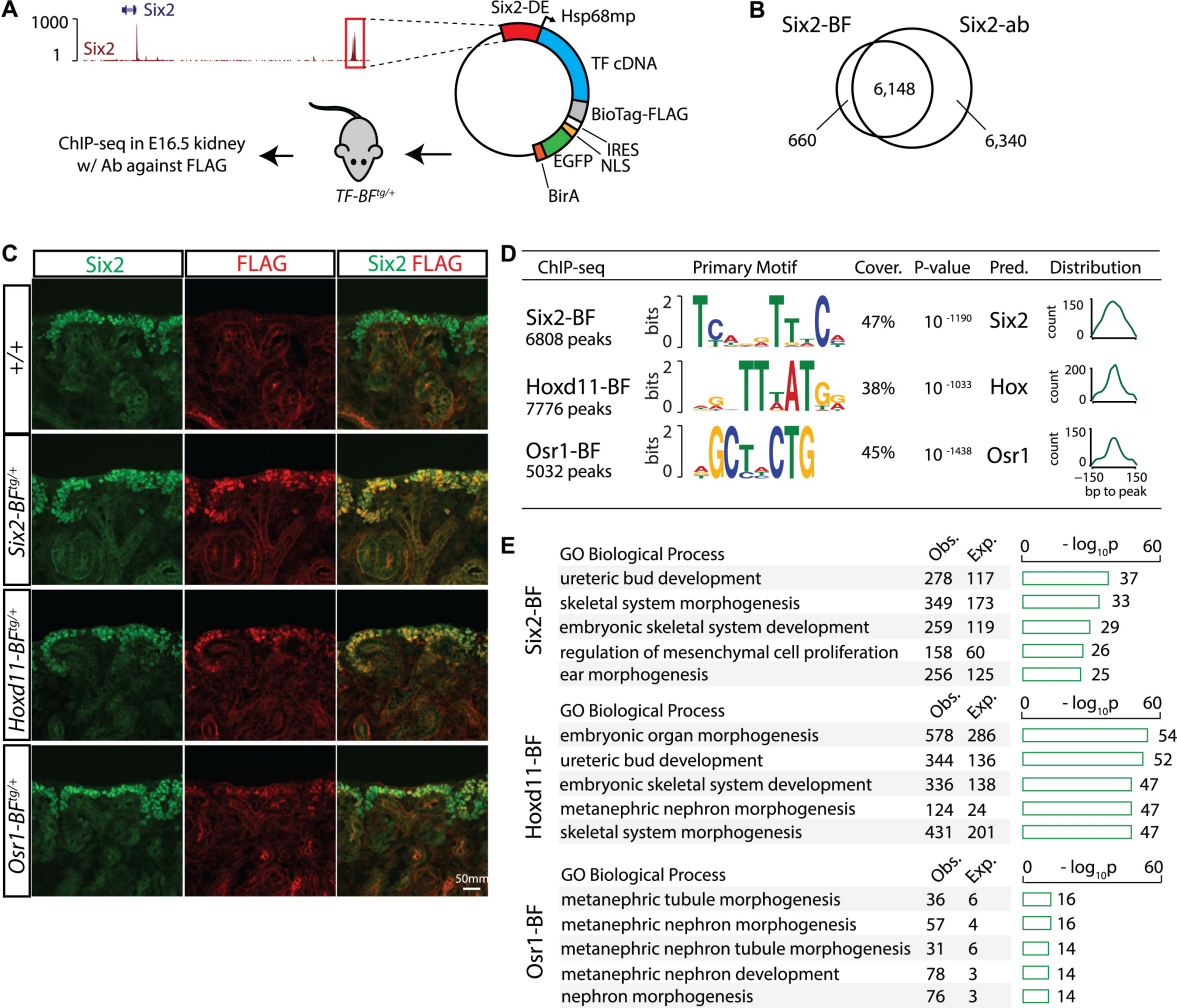
Identification of Six2, Hoxd11 and Osr1 binding sites in nephron progenitors by ChIP-seq. (A) Schematics shows characteristics of the transgenic mice used to generate nephron progenitor-specific ChIP-seq data: the Six2 distal enhancer (*Six2-DE*) drives nephron progenitor-specific expression of a FLAG-tagged transcription factor in the embryonic kidney. The IRES allows co-expression of GFP-BirA. Transgenic founders are utilized for FLAG ChIP-seq to identify progenitor specific programs. (B) Venn diagram shows overlap of peaks from FLAG ChIP-seq generated from *Six2-BF*^*tg/+*^ (Six2-BioTagFLAG) mouse kidneys and from the Six2 antibody (Six2-ab) ChIP-seq. (C) Immunostain for Six2 and FLAG at E16.5 shows the overlap of the two proteins for each transgenic line. (D) Motifs identified from Six2-BF, Hoxd11-BF, and Osr1-BF peaks with MEME (Multiple EM for Motif Elicitation; +/- 50 bp window). Coverage and p-values were calculated with FIMO (Find Individual Motif Occurrences) results. Smoothened histogram indicates distribution of motif-peak distance. Predicted TF = predicted transcription factor binding the discovered motif. (E) Functional annotation of Six2-BF, Hoxd11-BF, and Osr1-BF peaks performed using GREAT (Genomic Regions Enrichment of Annotations Tool). The top over-represented gene ontology terms belonging to the two categories are shown. ‘Obs.’, number of peaks associated with genes annotated with corresponding term; ‘Exp.’, number of peaks expected to be associated with genes annotated with corresponding term by chance. The barplot indicates the binomial p-values measuring over-representation of the corresponding terms.

To extend our understanding of the transcriptional regulatory networks operating within mouse nephron progenitors, we developed a transgenic approach to overcome the limited availability and inconsistency of working antibodies for key transcriptional components, and complications that arise from the diverse expression of regulatory factors elsewhere in the kidney. In this transgenic strategy, an epitope-tagged transcription factor-of-interest is expressed exclusively within the nephron progenitor compartment using a *Six2* distal enhancer (DE) previously shown to recapitulate *Six2*-like, nephron progenitor restricted expression (Fig 1A [28]). This approach obviates the need to enrich for the progenitor population in whole kidney samples simplifying ChIP procedures and avoiding potential artifacts introduced by tissue dissociation and fluorescence-activated cell sorting (FACS). We also took advantage of established tagging methods which have been utilized to successfully isolate protein:DNA complexes[38–41]. Each transcription factor-of-interest is appended with a BioTag-FLAG (BF) epitope at the C-terminus of the target protein. Co-production of an EGFP-BirA enzyme on the transgene through an IRES element also allows both ready visualization of transgenic kidneys and biotinylation of the biotin-recognition motif (BioTag) enabling an additional mode of isolation of factor-associated DNA or protein complexes through streptavidin affinity purification (Fig 1A). Though the biotin tagging strategy proved successful (S1C Fig) and provided a secondary purification option, we did not utilize it for any ChIP experiments as anti-FLAG antibodies were sufficient for all of the studies presented here.

To rigorously assess the efficacy of this strategy and to develop a protocol for whole kidney ChIP, we first generated *Six2-BF*^*tg*^ mice to determine whether Six2 ChIP-seq generated with the transgenic line (Six2-BF) replicates Six2 ChIP-seq using a Six2-specific antibody (Six2-ab) [3]. Six2-BF was restricted to the Six2+ nephron progenitors as indicated by specific detection of the anti-FLAG epitope (Fig 1C). FLAG ChIP-seq from Six2-BF+ kidneys identified 6808 Six2-associated regions in the Six2-BF data, with 90% of these peaks overlapping with Six2-ab peaks (Fig 1B). The two datasets were relatively correlated (R^2^ = 0.69) and, as expected, overlapping peaks were ranked higher than Six2-BF unique peaks indicating the variability in the data reflects marginal peak calls (S1A Fig). The most enriched motif discovered from the top 1000 peaks in the Six2-BF ChIP-seq dataset (‘TCANGTTTCA’, 47%, p-value = 10^−1190^, Fig 1D) matched and agreed with the identified Six2 motif from our previous ChIP studies (S1B Fig; [3, 28, 29]). The motif was relatively centered within the peaks suggesting direct binding of Six2 to the motif (Fig 1D). Additionally, electrophoretic mobility shift assays (EMSA) utilizing recombinant Six2 and a Six2 motif identified within the *Six2-DE* showed a strong interaction of Six2 protein with its DNA target (S2A Fig). Mutational analysis on this Six2-binding site demonstrated that the most conserved bases in the consensus (1T, 6T and 9C) were critical individually for effective protein-DNA interaction (S2A Fig). Wt1 and bHLH recognition motifs were also significantly enriched in Six2 binding regions (S1F Fig) consistent with an expected role for Wt1 within the progenitor compartment [31, 42], and an unidentified role for a bHLH factor. To interrogate the regulatory functions of Six2-BF, we performed GREAT Gene Ontology (GO) analysis [43] on Six2-BF peaks. Six2-BF peaks were highly enriched near genes associated with kidney development as reflected by the top GO term ‘ureteric bud development’ (Fig 1E).

In summary, the FLAG transgenic strategy robustly reproduced Six2-ab ChIP-seq data generated from wild-type kidneys identifying expected Six2 target and gene associations. These whole kidney-derived datasets significantly extend the depth of Six2 ChIP-seq peaks identified from earlier reports ([28]: 3907 peaks, [29]: 4306 peaks). While our transgenic strategy is useful for targets for which there are no working antibodies or when a progenitor-specific ChIP is desired, expression levels of the tagged protein or affinities of the FLAG antibody versus protein-specific antibodies (if one exists) may affect the number of relevant peaks discovered. Interestingly, although peaks identified uniquely with the Six2-ab showed lower levels of enrichment, these peaks still enriched for the Six2 motif at a similar level (46%) and were linked to kidney development GO terms suggesting a biological relevance to the interactions (S1A Fig). As nearly all Six2-BF peaks are contained within the larger Six2-ab dataset (~90%, Fig 1B), for a more complete analysis of Six2 bound target regions we used the latter dataset for subsequent analyses.

Having validated the transgenic strategy for generation of nephron progenitor specific ChIP-seq data, we established additional transgenic mouse lines to identify regulatory interactions mediated by other transcriptional regulators in nephron progenitors. Viable and phenotypically normal founders were generated for *Hoxd11* (*Hoxd11-BF*^*tg*^) and *Osr1* (*Osr1-BF*^*tg*^). Immunostaining with anti-FLAG antibodies confirmed the restriction of Hoxd11-BF and Osr1-BF to Six2+ nephron progenitors and validated the use of both transgenic lines for ChIP-seq analyses (Fig 1C). We also attempted to generate transgenic lines for Wt1, Hoxa11, Pax2, Sall1, and Eya1 but were unsuccessful in producing any founder animals. Further, we were not able to obtain transgenic progeny which survived past birth from the original Hoxd11-BF founder. These observations suggest transgene and/or transgenic line dependent lethality (see Discussion).

To map Hoxd11- and Osr1-associated genomic regions within nephron progenitors, we performed FLAG ChIP-seq on E16.5 *Hoxd11-BF*^*tg/+*^ and *Osr1-BF*^*tg/+*^ kidneys identifying 7776 Hoxd11-BF and 5032 Osr1-BF associated regions (Fig 1D). Osr1-BF protein levels were markedly lower and this may account for the lower number of target sites identified (Fig 1C). Both Hoxd11-BF and Osr1-BF peaks showed typical enhancer features: similar to the Six2-BF dataset the majority of the peaks were located >5kb from the transcription start site (TSS) within intronic (Six2-BF:46.6%, Hoxd11-BF: 46.2%, Osr1-BF: 44.7%) or intergenic regions (Six2-BF: 46.5%, Hoxd11-BF: 48.7%, Osr1-BF: 43.6%) (S1D and S1E Fig). GREAT analysis identified an enrichment for both factors near genes associated with processes related to metanephric kidney development (Fig 1E).

Using the same workflow adopted above for analysis of Six2 interactions, we identified the top DNA motif enriched in Hoxd11-BF (‘TTTATGG’, 38%, p-value = 10^−1033^, Fig 1D) and Osr1-BF datasets (‘GCTNCTG’, 45%, p-value = 10^−1438^, Fig 1D). Both motifs were well-centered within each peak dataset (Fig 1D). Multiple Hox factors are expressed in nephron progenitors and each may exhibit distinct binding preferences. While the predicted Hoxd11 motif has a prominent AT-rich Hox factor consensus feature, the motif differs from that identified through protein-DNA binding microarray (PBM) studies *in vitro* (‘TTTACGA’, [44], S2B Fig). EMSA analysis confirmed Hoxd11 binding and the relative importance of the bases 2T and 4A which are conserved in both the PBM and ChIP-seq based predictions, while the 1T and the 5T/C positions, which differed between the two predicted motifs, were not important for binding *in vitro* (S2B Fig). The Osr1 motif identified from our ChIP-seq data closely resembled that predicted from PBM studies (‘GCTACTG’, [44]) though no strong preference for the 4^th^ nucleotide position was seen in the *in vivo* motif. EMSA demonstrated Osr1 bound to the predicted Osr1 binding site within the *Six2-DE* (GCTGCTG). Interestingly, substituting an A in the 4G position to more closely reflect the PBM motif enhanced the Osr1 interaction (S2C Fig). These findings suggest that *in vivo* regulatory processes may prefer weaker binding, potentially adding greater flexibility to transcriptional interactions. Wt1 and bHLH motifs were also enriched in each peak dataset, as was observed for Six2-BF peaks (S1F Fig).

### Six2, Hoxd11, Osr1 and Wt1 co-bound sites predict key enhancers and targets of the nephron progenitors

A Wt1-like binding motif was predicted within all three datasets suggesting Wt1 co-regulation within Six2, Hoxd11 and Osr1 transcriptional networks. Other groups have published Wt1 ChIP from the whole embryonic kidney or glomerulus [30–32] but no nephron progenitor-specific Wt1 data has been generated. We attempted to generate a viable Wt1-BF transgenic line but failed, so we adopted a recently developed protocol for enriching nephron progenitors by magnetic-activated cell sorting (MACS) [45], and performed ChIP-seq with a Wt1-specific antibody on E16.5 nephron progenitors (Wt1-NP, S3A Fig).

Compared to a Wt1 ChIP from the whole kidney (Wt1-kidney) which we generated from the same stage (S3A Fig), the recovered motif from the Wt1-NP dataset, ‘CCTCCCCCNC’, closely matches the motif identified in our own whole kidney dataset, and published non-nephron progenitor-restricted Wt1 kidney ChIP data (S3B Fig, [30–32]). The motif also matched the predicted Wt1 motif that was highly enriched in the earlier Six2, Osr1, and Hoxd11 datasets (S1F Fig). The motif was centered in the ChIP peak dataset supporting direct DNA binding (S3B Fig). The nephron progenitor-specific Wt1 ChIP shared >50% of peaks with our whole kidney dataset. Shared target genes with roles in kidney development were focused on genes involved nephron progenitor maintenance and differentiation, while those unique to the whole kidney also targeted genes associated with podocytes (S3F Fig). This suggests that our Wt1-NP ChIP is representative of regulatory functions for Wt1 within nephron progenitors. The majority of peaks showed an intergenic (35%) and intronic (33%) distribution (S3D Fig). However, Wt1 showed significant enrichment near promoters within 5kb of the TSS (25%, S3C and S3D Fig), significantly more promoter enrichment than observed with the other factors (between 3.1 and 5.3%, S1E Fig), potentially reflecting Wt1’s binding preference to a cytosine-rich motif and GC enrichment at promoters. This result is in contrast to the Wt1 ChIP-seq performed by Motamedi et al., who found peaks to be enriched more distally [31]. However, if we performed GREAT analysis with the ‘basal plus extension’ parameter which includes larger regulatory domains compared to the more restricted ‘single nearest gene’ parameter which was utilized in all of our analyses, we observe a greater enrichment for Wt1-NP peaks 50-500kb from the TSS (S3C Fig). Importantly, the GREAT parameters recovered ‘ureteric bud development’ and ‘metanephric nephron morphogenesis’ terms which are consistent with Wt1 kidney functions (S3E Fig).

**Fig 2:**
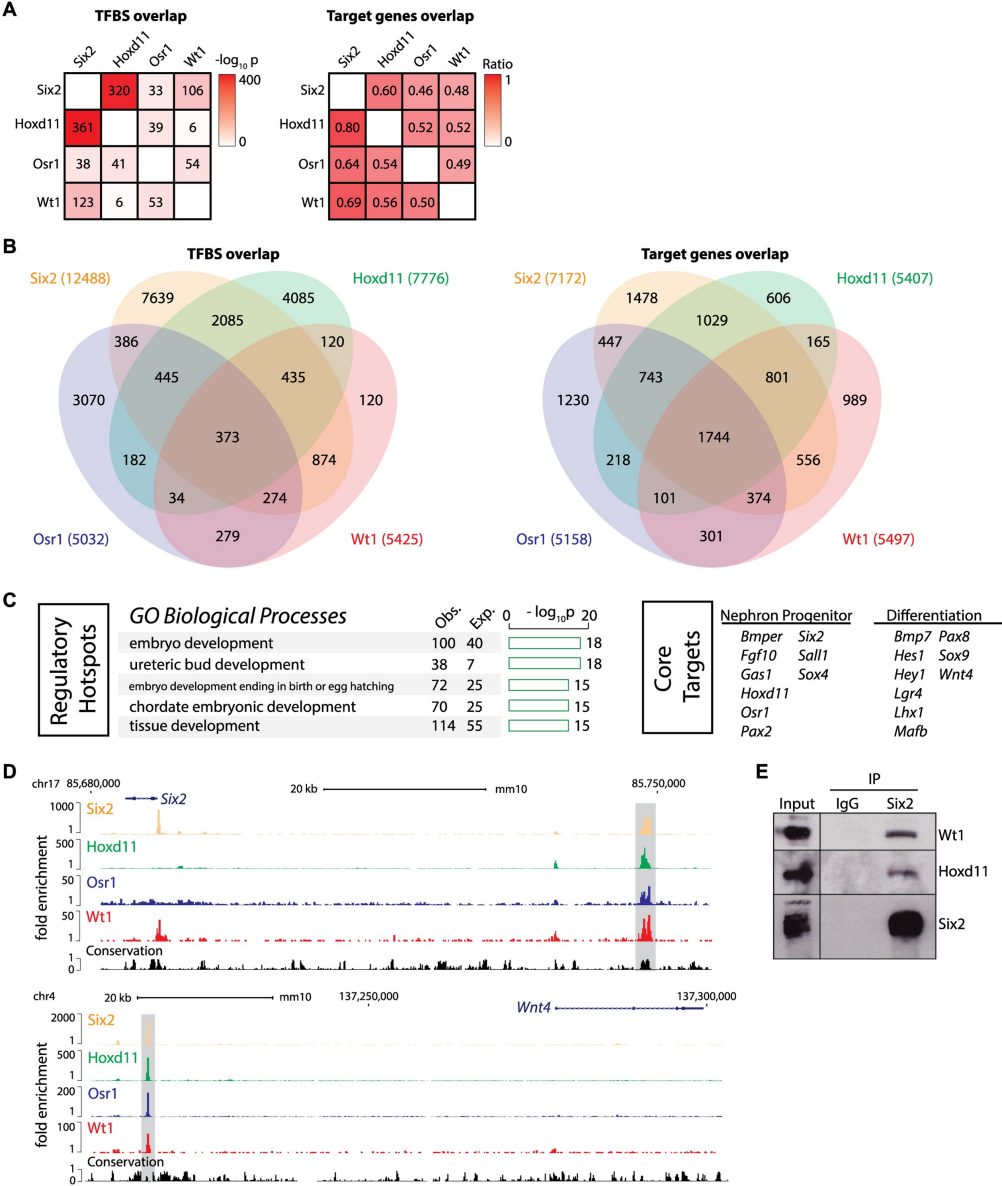
Regulatory hotspots in nephron progenitors defined by co-binding of Six2, Hoxd11, Osr1 and Wt1. (A) Heatmap shows significance of pairwise overlap between transcription factor binding sites (left, represented by binomial -log10 p-value) or between assigned target genes (right, represented by ratio). TFBS = transcription factor binding site. (B) Venn diagram shows the overlap of Six2, Hoxd11, Osr1, and Wt1 binding sites (left) and target genes (right). The 4-way overlapping sites were defined as the ‘regulatory hotspots’. The 4-way overlapping target genes were defined as ‘core targets’. (C) Barplots show result of gene ontology (GO) analysis on the ‘regulatory hotspots’ (left). Examples of ‘core targets’ known to have roles in the nephron progenitors and their differentiation are listed (right). (D) Genome browser view of Six2, Hoxd11, Osr1, and Wt1 ChIP-seq signals at the ‘regulatory hotspots’ (shadow area) near *Six2* and *Wnt4*. (E) Six2 immunoprecipitation from E16.5 kidney nuclear extracts. Western blot was probed with antibodies to Six2, Hoxd11, and Wt1 to identify protein complexes.

To investigate potential co-operative actions of Six2, Hoxd11, Osr1, and Wt1 in nephron progenitors, we analyzed all pairwise overlaps of transcription factor binding sites, and evaluated the statistical significance of such two-factor overlap. Not all genome fractions are accessible to transcription factor binding, and binding of many transcription factors correlates with open chromatin [46]. For simplicity, our statistical analysis is built on the assumption that only open chromatin, identified by utilizing the Assay for Transposase Accessible Chromatin with high-throughput sequencing (ATAC-seq) within nephron progenitors (see Methods for details of the approach and access to data), is accessible to any of the DNA binding factors analyzed in the current study. We found that the greatest significance of co-binding is observed between Six2 and Hoxd11 (-log_10_p = 320 at all Six2 sites where Hoxd11 is bound and -log_10_p = 361 at all Hoxd11 sites where Six2 is bound), and Six2 and Wt1 (-log_10_p = 106 at all Six2 sites where Wt1 is bound and -log_10_p = 123 at all Wt1 sites where Six2 is bound) interacting regions. The weakest co-association is between Wt1 and Hoxd11 (-log_10_p = 6 for each pairwise association), although still significant (Fig 2A). Potential target genes for each factor (based on GREAT analysis, [43]) were also subjected to pairwise comparisons. Hoxd11, Osr1, and Wt1 share the majority of their target genes with Six2 (ratio greater than 0.60 or 60%, Fig 2A and 2B). Hoxd11 shows the greatest overlap with Six2 (0.80 or 80%), although all pairwise overlaps showed that nearly half of the comparators target genes are shared with any one factor. These results suggest that these factors likely cooperate in regulatory actions within nephron progenitors.

Next, we overlapped all four datasets to identify sites where all factors converge in the potential regulation of target genes. We recovered 373 putative cis-regulatory modules where Six2, Hoxd11, Osr1, and Wt1 associated within 1kb of each other (Fig 2B, S1 Table). Regions co-bound by all four factors displayed the strongest Six2 binding. In addition, Six2 peaks bound by any three-factor combination were on average stronger than two-factor combinations, while Six2 peaks bound by any factor in combination with Six2 were stronger than Six2-only peaks (S1H Fig).

We refer to regions co-bound by all four factors as ‘regulatory hotspots’ hypothesizing that these may play a key role in nephron progenitor programming. Consistent with this view, regulatory hotspots were enriched around genes annotated to developmental processes such as ‘ureteric bud development’ (Fig 2C). Further, two regulatory hotspots are known from published studies to drive transgenic reporters with expression profiles reflecting the putative target genes: a region ~60kb upstream of *Six2* which corresponds to the *Six2-DE* used in our transgenic strategy and the *Wnt4*-*DE* 50kb upstream of *Wnt4* (Fig 2D, [28]). Sall1 is also bound at the *Six2* distal enhancer but not at the *Wnt4* enhancer site[29]. *Six2* is largely restricted to the nephron progenitors while *Wnt4* expression is absent from nephron progenitors but activated on progenitor induction in the formation of differentiating renal vesicles [2, 47]. Thus, engagement of the four factors can occur on target genes for nephron progenitors or genes activated shortly after the onset of nephrogenesis. Other putative targets of regulatory hotspots include *Fgf9* which is expressed by nephron progenitors and is involved in regulating their maintenance [48], and *Pax8* which regulates nephron progenitor differentiation [49] (S1 Table). *Tsc22d1* and *Mgat5* also represent putative targets and knockouts of these genes are reported to generate kidney phenotypes [50, 51] (S1 Table).

Regulatory information may also converge on a common target through alternative enhancer usage. To examine this possibility, we intersected the predicted target gene sets for each factor and identified 1744 genes sharing Six2, Hoxd11, Osr1, and Wt1 associated peaks (Fig 2B, S2 Table). The set of genes identified as having all four factors co-associated at one putative cis-regulatory module or dispersed through multiple interactions sites are predicted to define a set of genes with a significant role in nephron progenitors or their derivatives; we termed this group ‘core targets’ (Fig 2C, S2 Table). This set includes genes expressed in nephron progenitors and implicated in progenitor maintenance and self-renewal including *Six2*, *Pax2*, *Sall1*, *Sox4*, and *Gas1* [2, 19, 52–54]. However, the ‘core targets’ also included genes normally activated downstream in the induced/developing nephron such as *Wnt4*, *Lhx1*, *Pax8*, *Hes1*, and *Irx1/2* [47, 49, 55–57].

To determine whether interactions amongst these transcription factors exist *in vivo*, we performed immunoprecipitations with Six2 antibodies from E16.5 kidney nuclear lysates. Six2 was able to co-immunoprecipitate Hoxd11 and Wt1 (Fig 2E); however, the absence of a working Osr1 antibody precluded analysis of this factor although recent studies show Six2 and Osr1 complex *in vitro* [17]. Six2 is also purported to complex with Sall1 [29] though we could not replicate this interaction with available antibodies in our assay. Taken together, these data provide evidence for endogenous, multi-protein complexes among three of the four factors.

### Transcription factor co-binding is preferentially associated with genes active in differentiating structures and reveals novel targets

**Fig 3:**
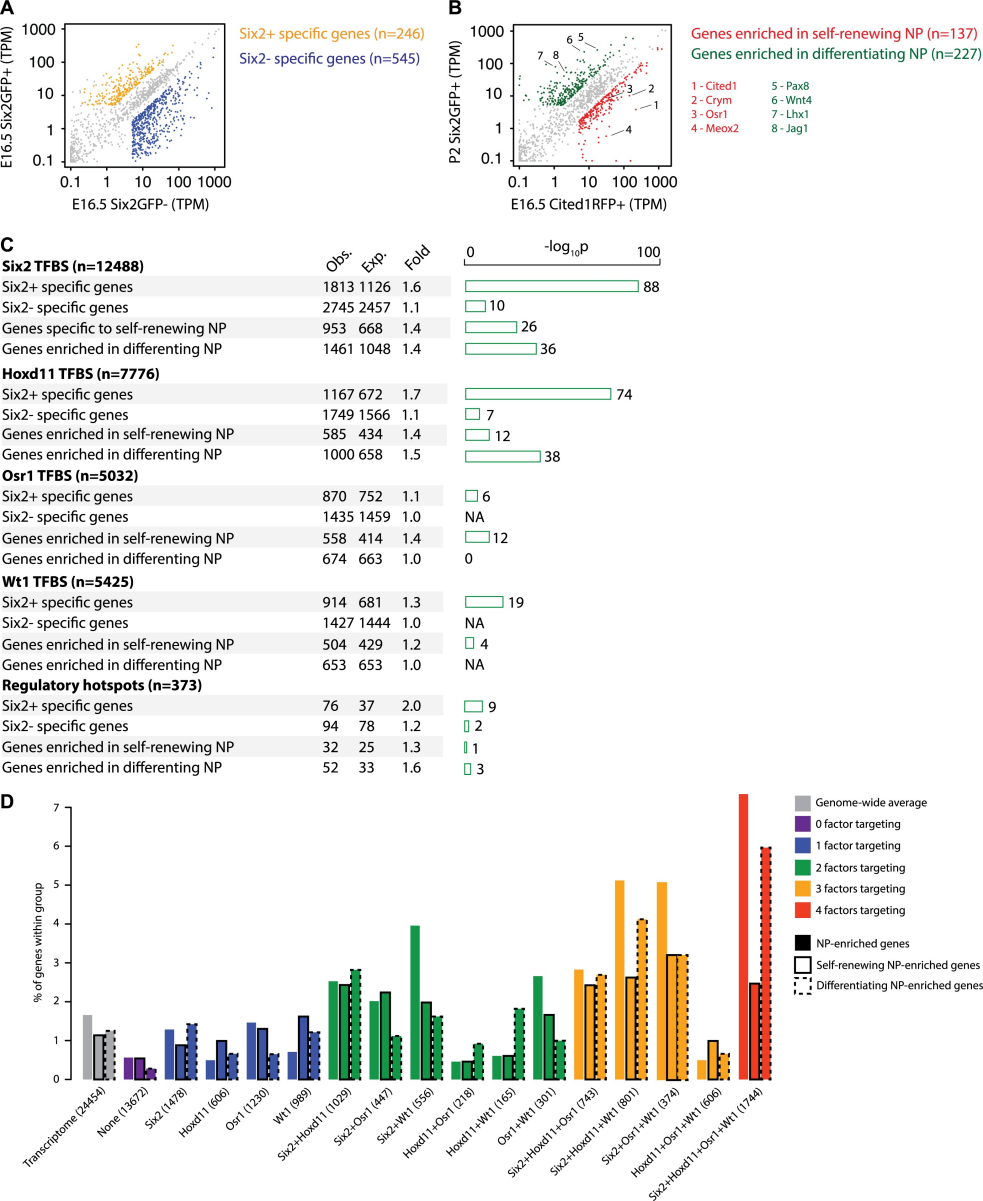
Six2, Hoxd11, Osr1, and Wt1 binding sites are enriched near nephron progenitor specific genes and those associated with differentiation programs. (A) Scatter plots show gene expression profiles and correlation of the Six2GFP+ versus the Six2GFP- RNA-seq from E16.5 mouse kidney cortex. Specific genes for each category are highlighted in orange (Six2+) or blue (Six2-). TPM = Transcripts Per Kilobase Million. (B) Scatter plots show gene expression profiles and correlation of RNA-seq from E16.5 Cited1RFP+ cells versus P2 Six2GFP+ cells. Genes specific to each population are highlighted in red (Cited1RFP+) or green (Six2GFP+). Examples of specific genes are listed and highlighted on the plot. (C) Barplots show p-values indicating enrichment of Six2, Hoxd11, Osr1, and Wt1 binding sites, as well as regulatory hotspots (Six2/Hoxd11/Osr1/Wt1 overlapping sites) in genes that are specific to the Six2+ cortex fraction, specific to the Six2- cortex, enriched in self-renewing nephron progenitors, or enriched in differentiating nephron progenitors, respectively. The regulatory domain was defined as +/-500 kb from transcription start site. TFBS = transcription factor binding site. ‘Obs.’, number of peaks associated with genes annotated with corresponding term; ‘Exp.’, number of peaks expected to be associated with genes annotated with corresponding term by chance. Fold represents the fold enrichment or expected values. (D) Bar plots showing the percentage of total genes for each condition (x-axis) that falls into each category of 1) nephron progenitor (NP) enriched, 2) enriched in self-renewing nephron progenitors, or 3) enriched in differentiating progenitors.

We sought to identify whether Six2, Hoxd11, Osr1, and Wt1 are each involved in activating or repressing gene expression in nephron progenitors. First, we generated RNA-seq expression profiles of E16.5 *Six2TGC*^*tg/+*^ kidney cortex preparations FAC-sorted for GFP+(Six2+) or GFP-(Six2-) cells. Six2+ cells would represent the nephron progenitor population (both self-renewing and recently induced) and Six2- cells would largely represent stromal cells as well as ureteric bud tip cells and endothelial cells. Genes with a TPM (Transcripts Per Kilobase Million) value >5 and a fold difference >3 between the two cell types were identified: 246 genes were enriched in the Six2+ fraction and 545 genes were enriched in the Six2- cortex fraction (Fig 3A, S3 Table). We asked whether ChIP-seq peaks of any of the transcription factors or the regulatory hotspots are preferentially located adjacent to differentially expressed genes. The results show that peaks from all ChIP-seq datasets occur significantly more often around genes enriched in the Six2+ cells (Fig 3C) consistent with a specific role in regulating the nephron progenitor cell versus other cell types of the kidney cortex. However, regulatory hotspots near *Foxd1*, a marker of self-renewing stromal progenitors [58], and *Wnt11*, a ureteric tip marker required for normal kidney development [59] (S1 Table), raises the possibility that the four factors may also work together to repress these genes within nephron progenitors.

Nephron progenitor cells can be divided into Cited1+/Six2+ self-renewing progenitors and Cited1-/Six2+ differentiating progenitor cells [60]. To address the relationship between regulatory hotspots and programs of progenitor maintenance or commitment, we performed RNA-seq analysis to identify progenitor-specific and early induction gene sets. For the former, a transcriptional profile was generated for E16.5 Cited1+; RFP+ cells from *Cited1-nuc-TagRFP-T*^*tg/+*^ kidneys while Six2+; GFP+ cells from *Six2TGC*^*tg/+*^ P2 kidneys were used to generate the latter dataset (S4 Table, [61]). As expected, *Cited1* levels were appreciably lower in the P2 Six2+ cells (200.9 TPM in E16.5 Cited1+ cells vs. 3.8 TPM in P2 Six2+ cells) while *Wnt4* transcripts were markedly increased (9.0 TPM in E16.5 Cited1+ cells vs. 219.1 TPM in P2 Six2+ cells) supporting our classification of these datasets (S4 Table).

As expected, a comparison of the genes with a TPM >5 and a fold difference >3 between the two cell types showed self-renewing nephron progenitor-specific genes such as *Cited1* and *Osr1* enriched in the E16.5 Cited1+ cell dataset whereas genes involved in progenitor differentiation such as *Pax8* and *Wnt4* were enriched in the P2 Six2+ cell dataset (Fig 3B, S4 Table). Six2 and Hoxd11 displayed similar enrichment near genes up-regulated in either self-renewing nephron progenitors or in differentiating progenitors (1.4–1.5 fold; Fig 3C) consistent with roles in promoting the progenitor state, and either preventing or priming nephron forming programs. Osr1 and Wt1 interactions were slightly enriched near genes associated with self-renewing nephron progenitors (1.4-fold vs 1.0-fold for Osr1, 1.2-fold vs 1.0-fold for Wt1; Fig 3C). Interestingly, the regulatory hotspot associated gene lists showed a higher enrichment around genes upregulated in differentiating cells versus self-renewing progenitors (1.6-fold vs. 1.3-fold; Fig 3C).

Next, for each single factor or combination of factors we compared the percent of target genes in distinct transcriptional categories: nephron progenitor enriched (E16.5 Six2+ cells), self-renewing nephron progenitor enriched (E16.5 Cited1+ cells), or differentiating nephron progenitor enriched (P2 Six2+ cells) relative to the whole transcriptome. Target genes unique to any single factor were not enriched in any of these categories (≤1.6% for each) compared to the whole transcriptome (≤1.6% for each) suggesting that single factor input has no particular relevance to nephron progenitor function. Similar observations hold when Hoxd11 co-targeting is examined with Osr1 and Wt1, (≤1.8%). but not with a Six2 binary combination (≥2.5%) suggesting that Hoxd11 has a strong preference for co-regulation of target genes with Six2 (Fig 3D). Generally, the greatest enrichments are observed when all four factors are bound near the target gene in any category (2.4–7.2%) consistent with co-regulatory input by multiple factors impacting target gene regulation to the greatest extent. In agreement with our earlier analyses, the four-factor overlap has a preference for genes expressed upon differentiation rather than in self-renewing progenitors (5.9% compared to 2.4%; Fig 3D).

While we have described target genes with known functions in kidney development, we wanted to identify potentially novel candidate genes which are targets of co-regulation either by one cis-regulatory module or dispersed through multiple interactions sites. Target genes of interest include *Shisa2* and *Shisa3* which are enriched in self-renewing nephron progenitors (S2 Table). *Shisa2* is a modulator of Wnt and Fgf signaling, specifically attenuating such signals. The majority of mutant mice exhibit dwarfism and half die postnatally. *Shisa3* is a related family member although no overt phenotype was observed for the null allele [62]. *Pdgfc* and *Pdgfa* are enriched in the self-renewing and differentiating progenitors, respectively (S2 Table). Their conserved expression in these cell populations of developing mouse and human kidneys have been reported [63–65]. *Pdgfa* and *Pdgfc* double mutants have a reported deficiency in cortical renal mesenchyme, however, the mutant kidney phenotype was not analyzed in detail [66]. *Ccnd1* (cyclin D1) is a putative target that shows a nearly 7-fold increase in expression in P2 Six2+ cells versus E16.5 Cited1+ cells (S2 Table). *In situ* hybridization confirms strong *Ccnd1* in E15.5 pretubular aggregates and early differentiating nephrons (www.gudmap.org, [67, 68]). This suggests that the regulatory networks may directly modulate cell cycle dynamics and balance progenitor proliferation or alternatively may prime putative enhancers of *Ccnd1* for rapid activation upon nephron progenitor induction. *Sema5a* and *Epha4* are predicted targets with a similar ~6-7-fold increase in expression in differentiating progenitors confirmed by *in situ studies* (S2 Table; www.gudmap.org, [67, 68]) suggesting factor regulation of targets genes controlling local cell interactions.

### Deletion of the *Six2* and *Wnt4* distal enhancers reveals their roles in modulating target gene expression

**Fig 4:**
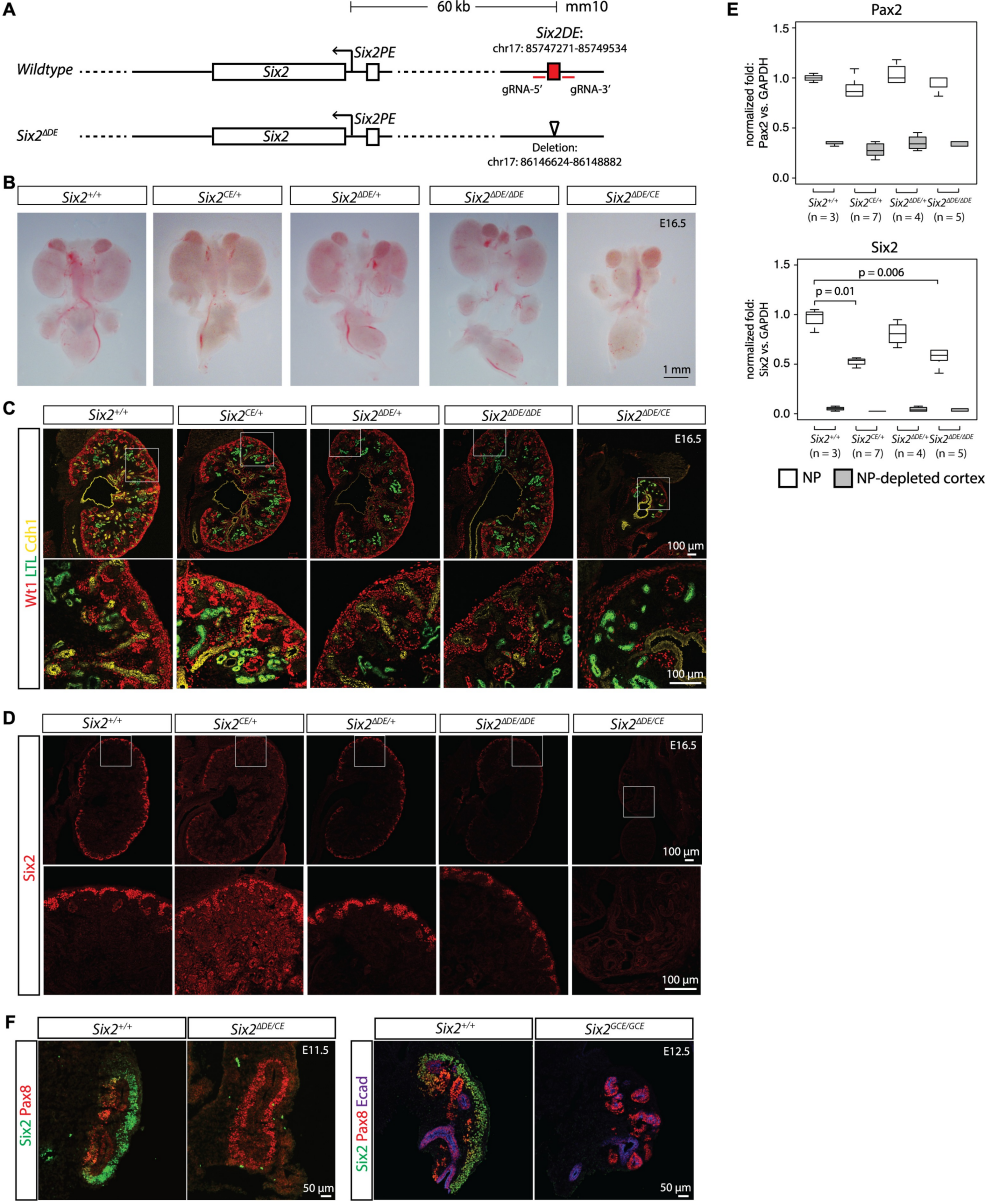
Deletion of the *Six2* distal enhancer leads to reduction in *Six2* levels and concomitant loss of a *Six2* allele results in severe renal hypoplasia. (A) Schematic of the *Six2* locus showing the location of the proximal (PE) and distal (DE) enhancer elements. The *DE* was targeted for deletion using CRISPR/Cas9 and the resulting Cas9-mediated deletion of the *Six2-DE* is shown. (B) Brightfield images of whole urogenital systems from E16.5 embryos resulting from *Six2*^*∆DE/+*^ matings or *Six2*^*CE/+*^ x *Six2*^*∆DE/+*^ crosses. (C) Immunostaining for Wt1 to identify nephron progenitors and podocytes, LTL (*Lotus tetragonolobus* lectin) to mark proximal tubules, and Cdh1 to show the collecting duct network of kidneys associated with (B). (D) Immunostaining for Six2 to identify nephron progenitors in kidneys associated with (B). (E) Box plots showing results of qPCR for *Six2* and *Pax2* (normalized to *GAPDH*) from nephron progenitors (NP) and nephron progenitor-depleted cortex. Genotypes and number of samples analyzed are shown. (F) Samples from *Six2*^*GCE/GCE*^ were compared to *Six2*^*∆DE/GCE*^ collected at early stages of kidney development and immunostained with Six2 to mark the nephron progenitors, Pax8 to identify differentiating structures (Pax8 antibody appears to cross react with Pax2 as seen by expression in Ecad+ collecting duct), and Ecad to mark epithelial structures.

**Fig 5:**
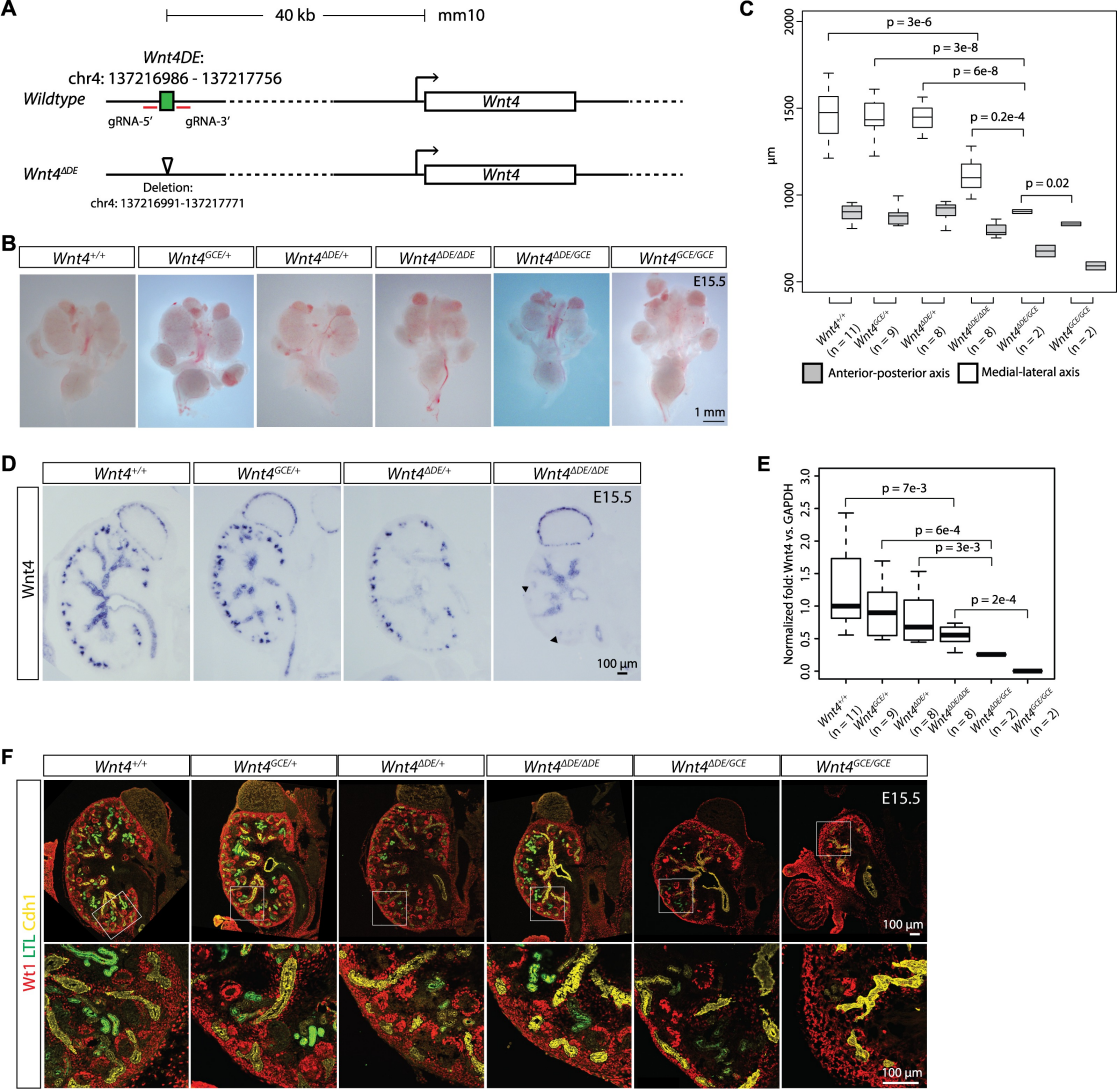
Deletion of the distal enhancer for *Wnt4* results in reduced expression of *Wnt4* specifically in renal vesicles and smaller kidneys. (A) Schematic of the *Wnt4* locus showing the location of the distal enhancer (DE) element. The DE was targeted for deletion using CRISPR/Cas9 and the resulting Cas9-mediated deletion of the *Wnt4-DE* is shown. (B) Brightfield images of whole urogenital systems from E15.5 embryos. (C) Measurements of the anterior to posterior axis and medial to lateral axis of kidneys representing the genotypes shown in (B). (D) Section *in situ* hybridization for *Wnt4* on kidneys from *Wnt4*^*∆DE/+*^, *Wnt4*^*∆DE/∆DE*^ and *Wnt4*^*GCE/+*^ embryos. (E) Box plots showing results of qPCR for *Wnt4* (normalized to *GAPDH*) from whole kidneys at E15.5. Genotypes and number of samples analyzed are shown. (F) Immunostaining for Wt1 to identify nephron progenitors and podocytes, LTL to mark proximal tubules, and Cdh1 to show the collecting duct network of kidneys associated with (B).

To examine the functional significance of ‘regulatory hotspots”, we focused on *Six2-DE* (chr17: 85747271–85749534; Fig 4A) and *Wnt-4 DE* (chr4:137216986–137217756; Fig 5A) elements previously verified in transgenic reporter assays [28]. To examine the requirement for each enhancer, we used CRISPR/Cas9 gene editing technology to delete each enhancer in B6SJLF1/J mice. The *Six2-DE* deletion and *Wnt4-DE* deletion were confirmed in founder lines by PCR and Sanger sequencing of products (*Six2*^*∆DE*^: chr17:85747284–85749542; *Wnt4*^*∆DE*^: chr4:137216991–137217771). For the *Six2-DE* knockout, we examined kidneys at E16.5 and observed no obvious difference in the size of wildtype, *Six2*^*∆DE/+*^ and *Six2*^*∆DE/∆DE*^ kidneys (Fig 4B). Six2+ and Wt1+ nephron progenitors were present in *Six2*^*∆DE/∆DE*^ kidneys though Six2 levels appear reduced relative to wild-type embryos (Fig 4C and 4D). Nephron structures were formed as reflected by the presence of podocytes and proximal tubules, labeled by Wt1 and LTL (*Lotus tetragonolobus* lectin), respectively (Fig 4C). The *Six2*^*∆DE/∆DE*^ mice were viable; no phenotype was observed.

To more accurately assess the effect of the distal enhancer deletion on *Six2* expression, we used qPCR to measure relative *Six2* levels in nephron progenitors of E16.5 kidneys. A 40% reduction of *Six2* mRNA was measured in *Six2*^*∆DE/∆DE*^ nephron progenitors compared to wildtype (Fig 4E, p-value = 0.006); higher levels than in mice heterozygous for a *Six2* null allele (*Six2*^*CE/+*^, [69]) where *Six2* transcripts were reduced approximately 50% relative to wild-type as expected (Fig 4E). The levels of *Pax2* mRNA, which is not dependent on Six2 [2], were relatively similar across all genotypes showing a Six2-specificity for the *Six2-DE* deletion. Strikingly, when *Six2* levels were further reduced by combining a *Six2*^*∆DE*^ allele with a *Six2* null allele (either *Six2*^*CE/+*^ or *Six2*^*GCE/+*^ [69]), the resultant *Six2*^*∆DE/CE*^ embryos exhibited severely hypoplastic kidneys at E16.5, with a complete absence of Six2+ nephron progenitors, mirroring the phenotype of complete removal of *Six2* activity (Fig 4B and 4D, [2]) where only a few glomeruli (Wt1+) and tubules (LTL+) have formed by E18.5 (S4 Fig). As early as E11.5, at the outset of active kidney morphogenesis, *Six2*^*∆DE/GCE*^ kidneys were devoid of Six2+ nephron progenitor cells but filled with Pax8+ differentiating nephron progenitors as in Six2 protein null mutant kidneys (Fig 4G, [2]). Taken together these results demonstrate that *Six2-DE* accounted for approximately 40% of *Six2* expression and by combining one *Six2-DE* allele with a *Six2* null allele, the remaining *Six2* mRNA levels (predicted to be 30% of wild-type levels) were insufficient for Six2-mediated maintenance of the nephron progenitor state.

Next, we investigated a ‘regulatory hotspot’ predicted to function in progenitor differentiation. Deletion of the *Wnt4* distal enhancer resulted in mutant kidneys that are ~25% smaller than those from wildtype animals (p = 0.2e-4; Fig 5B and 5C). Nephrons developed in *Wnt4*^*∆DE/∆DE*^ kidneys as reflected by presence of both LTL+ proximal tubules and Wt1+ podocytes (Fig 5D) and *Wnt4*^*∆DE/∆DE*^ mice are viable. Interestingly, *in situ* hybridization revealed that expression of *Wnt4* is significantly reduced in renal vesicles but remained largely unchanged in the renal medulla of *Wnt4*^*∆DE/∆DE*^ kidneys consistent with an overall reduction of *Wnt4* mRNA levels in *Wnt4*^*∆DE/∆DE*^ kidneys measured by qPCR (Fig 5F). Thus, the *Wnt4-DE* plays a functional role in regulating *Wnt4* mRNA levels in forming nephrons (Fig 5E). The *Wnt4*^*∆DE/∆DE*^ phenotype was less severe than Wnt4 protein null mutants where the severely hypoplastic kidney lacks nephron tubules and glomeruli [47]; indeed, low levels of *Wnt4* RNA were detected in *Wnt4*^*∆DE/∆DE*^ kidneys (Fig 5D; arrows in Fig 5E). When the *Wnt4*^*∆DE*^ allele was combined with a *Wnt4*^*GCE*^ protein null allele [69], *Wnt4*^*∆DE/GCE*^ kidney size and nephron structures were further reduced, though kidneys were still larger than *Wnt4* null kidneys (Fig 5B–5D) and *Wnt4* mRNA levels were markedly reduced in whole kidney PCR (Fig 5E and 5F). Taken together these results indicate a dose-dependent reduction in kidney size through reduced nephrogenesis upon decreasing *Wnt4* activity. Further, residual levels of *Wnt4* activity in *Wnt4*^*∆DE/GCE*^ kidneys were sufficient to drive low levels of nephrogenesis. Clearly, the *Wnt4-DE* plays a role in maintaining appropriate levels of *Wnt4* transcripts in the nephrogenic program to ensure a normal program of kidney development.

### The *Br* mouse is the result of an inversion altering the *Six2* regulatory landscape

**Fig 6:**
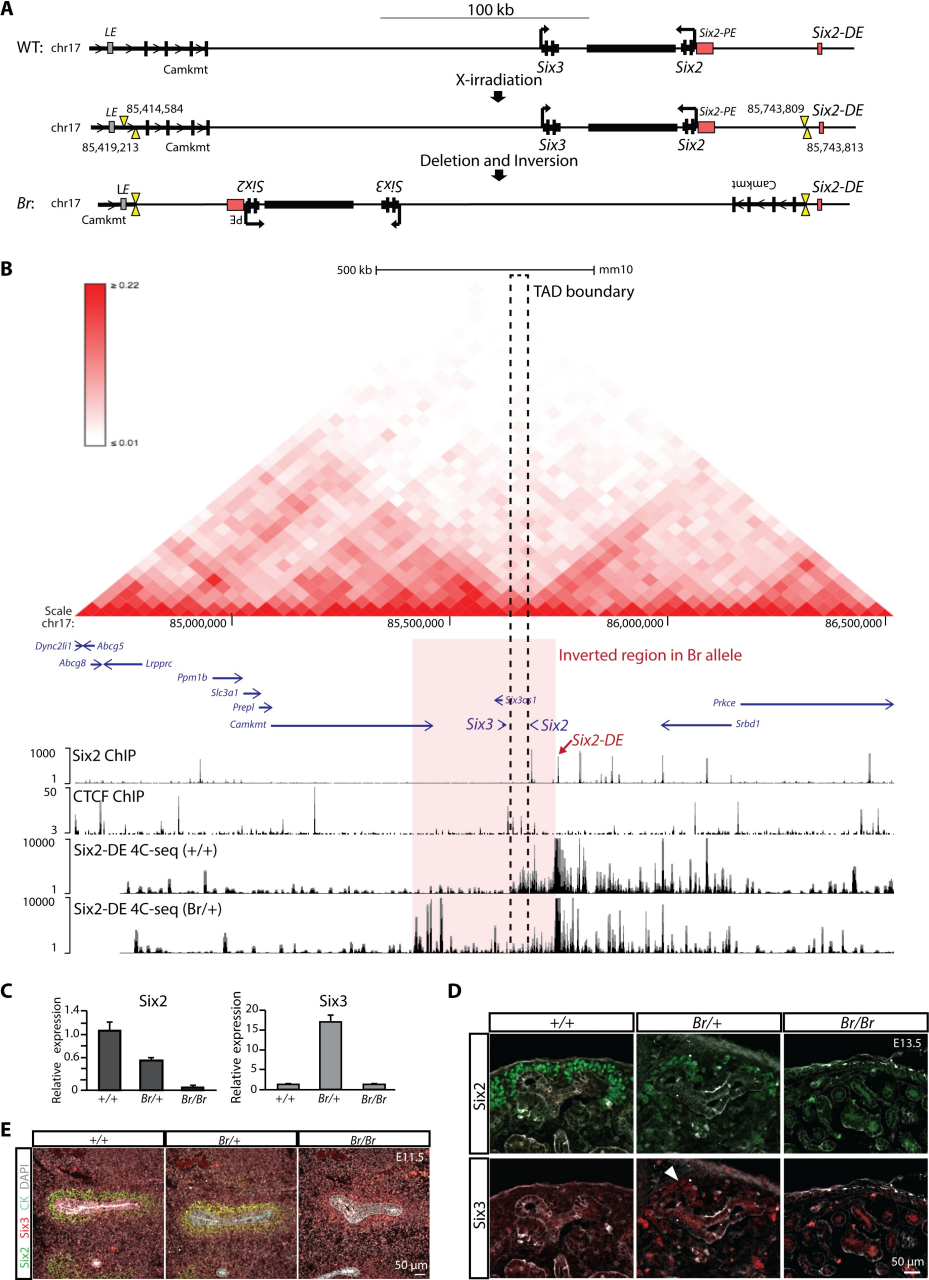
The *Six2* regulatory landscape is altered in the *Br* mouse leading to reduced Six2 expression and ectopic Six3 expression in the kidney. (A) Schematic showing the X-irradiation induced breakpoints and subsequent deletion with inversion that resulted in the *Br* allele. LE = Lens enhancer (putative), PE = proximal enhancer, DE = distal enhancer. Black box between the *Six2* and *Six3* loci represents the predicted boundary. (B) Interaction matrix (top) generated by Hi-C data (Hardison lab hESC Hi-C data, http://promoter.bx.psu.edu/hi-c/view.php). Genomic view showing Six2 ChIP-seq, CTCF-NP ChIP-seq, and 4C-seq data (bottom). The region inverted in the *Br* allele is highlighted. Dashed square indicates a predicted TAD boundary element that lies between *Six3* and *Six2* loci [70]. (C) qPCR showing the relative expression levels of *Six2* and *Six3* in E13.5 kidneys of the indicated genotype. (D) E13.5 kidneys of the indicated genotype were sectioned and immunostained for Six2 and Six3. Arrow points to the low level Six3 expression in nephron progenitors. (E) Immunostaining for Six2, Six3, cytokeratin (CK), and DAPI in E11.5 kidneys of the indicated genotype.

*Six2* lies ~60 kb from a related family member *Six3*, although significant *Six3* expression is not observed in the self-renewing nephron progenitors (S4 Table) indicating a specificity in *Six2-DE* interactions. Topologically associating domain (TAD) boundaries have been described as CTCF-enriched sites which serve as insulators and prevent promiscuous enhancer activity [70]. We performed CTCF ChIP-seq using E16.5 purified nephron progenitors to identify CTCF-bound regions of the genome (Fig 6B and S3G and S3H Fig). We identified strongly bound CTCF sites between the *Six2* and *Six3* locus, most of which are consistent with ENCODE data analyzing whole P0 kidney samples (S5B Fig, ENCODE experiment ENCSR143WOK, submitted by Richard Myers, HAIB, [71]) and predictions from Hi-C on mouse ES cells (TAD: chr17:85640660–85680660, S5A Fig, [70]). We hypothesize that this region serves as a TAD boundary to prevent the *Six2-DE* from engaging the *Six3* promoter. Consistent with this view, there is a marked bias in the engagement of regulatory factors in nephron progenitors to the *Six2* side of this putative boundary, 5’ to the *Six2* transcriptional start site (Fig 6B).

With these insights into regulation of *Six2*, our attention was drawn to the *Brachyrrhine* (*Br*) mouse, an X-irradiation induced mutant that displays kidney hypoplasia and frontonasal dysplasia, and maps to the *Six2* region of chromosome 17 [72]. Though *Br* mutants have significantly reduced *Six2* expression in the kidney and craniofacial tissues, no mutation has been found in the *Six2* transcription unit or within 1.8 kb upstream of the start codon which includes the *Six2-PE* elements [72]. Interestingly, *Six2* is ectopically expressed in the developing lens of *Br* heterozygous and homozygous animals, a normal site of *Six3* expression [72]. Given that irradiation induces large-scale genomic rearrangements, we speculated that the *Br* mutation led to a chromosomal rearrangement that removed *Six2* from enhancer(s) directing normal regulatory input to the nephron progenitor population, placing *Six2* under the control of *Six3* regulatory elements normally inaccessible the other side of a CTCF-dependent boundary element.

Next generation sequencing and sequence alignment identified the underlying sequence change in the *Br* mutant (S1 Supplemental Material and Methods). The main feature was a large inversion of 324,596bp including both the *Six2* and *Six3* loci. The inversion moves *Six3* ~206kb from the *Six2-DE*, actually further than in the wild-type organization, but importantly the inversion removes the intervening TAD boundary (Fig 6B). In contrast, *Six2* is repositioned on the other side of this boundary element within *Six3*’s unchartered regulatory territory (Fig 6B). In addition to the inversion, two small deletions were detected: a 4,630bp deletion (chr17:85414584–85419213) 5’ to the *Six3* TSS in the intron of *Camkmt*, and a 5bp deletion between the *Six2-PE* and *Six2-DE* (chr17:85743809–85743813, Fig 6A and 6B). The results from the sequencing and computational analysis were confirmed by allele-specific diagnostic PCR assays (S5C and S5D Fig). The inversion also separates the last 4 exons of *Camkmt* from the rest of the transcription unit. However, *Camkmt* has a TPM of only 2.26 in the E16.5 Cited1+ nephron progenitor cells and homozygous mutant mice are viable (International Mouse Phenotyping Consortium, http://www.mousephenotype.org/, Release 5.0 [73]), so *Camkmt* is unlikely to contribute to the kidney phenotype. The rearrangement predicts: i) ectopic *Six2* expression in *Six3’s* normal expression domain, the lens, as *Six3* enhancers can now target *Six2*, and ii) an abnormal interaction between the *Six2-DE* and *Six3* promoter resulting in ectopic *Six3* expression in nephron progenitors.

To directly examine interaction of *Six2-DE* with *Six2* and *Six3* promoters, we performed 4C-seq [74] using *Six2-DE* as the view point. As expected, in wildtype kidneys *Six2-DE* interacts with a broad region that includes *Six2* transcription start site (TSS) (Fig 6B), with the local maxima 7.2 kb upstream of Six2 *TSS*. Noticeably, *Six2-DE* interaction was restricted by the TAD boundary between *Six2* and *Six3* (Fig 6B and S5B Fig) and no interaction was observed around the *Six3* TSS. In kidneys from *Br/+* embryos, strong *Six2-DE* contacts were now observed in the segment of the inverted region that was repositioned between the *Six2-DE* and CTCF-bound TAD boundary element (Fig 6B and S5B Fig). As expected, with the loss of one wildtype allele in *Br/+* embryos, *Six2-DE* interactions with *Six2* TSS, and in general with the region on *Six2* side of the TAD boundary, were significantly reduced. A relatively strong, *de novo* interaction of the *Six2-DE* was observed ~15 kb upstream of *Six3* TSS (Fig 6B and S5B Fig) consistent with the model of *Six3* expression driven, at least in part, by the *Six2-DE* in the *Br* allele. Importantly, the predicted *Six2-DE/Six3* upstream contact in the *Br* allele occurs over a distance of 200 kb from the *Six2-DE* to the *Six3* TSS, a longer interval than the ~130 kb that separates these non-interacting elements in the wild-type allele (S5B Fig). Therefore, the differential interaction of *Six2-DE* with *Six2* TSS and *Six3* TSS between wildtype and *Br/+* cannot be attributed to shortened distance from *Six2-DE* to *Six3* TSS. Rather, this data supports specific regulation by *Six2-DE* to *Six2* and *Six3* that is defined by the TAD boundary.

As a result of the altered chromatin architecture introduce by the genomic inversion, ectopic *Six2* expression has been reported in the lens of *Br/Br* mutants [72], and *Six3* expression was reduced in this structure (S5E Fig). Quantitative PCR detected *Six3* expression in the kidneys of *Br/+* mice at E13.5 (Fig 6C) and Six3 protein was detected in Six2+ nephron progenitors (Fig 6D). *Br/Br* mutants resemble *Six2* null mutants and have no nephron progenitors at this stage [2] (Fig 6C and 6D). When *Br/Br* mutants were examined at E11.5, they showed a similar loss and premature differentiation of nephron progenitors as in *Six2* null mutants but interestingly low-levels of Six3 were detected in differentiating progenitors (Fig 6E). Ectopic Six3 was also observed in the cranial base of *Br* mutants at E14.5 concomitant with decreased Six2 levels (S5F Fig). Taken together these data lend additional weight to the importance of the *Six2-DE* in directing *Six2* expression and reveal higher order principles of topological organization acting in conjunction with this enhancer to provide target gene specificity to the regulatory landscape.

## Discussion

In this study, we utilized novel transgenic mouse strains to map the nephron progenitor-specific interactions of Six2, Hoxd11, and Osr1, and incorporated nephron progenitor-specific ChIP-seq profiling of Wt1, to identify the regulatory genome controlled by these four factors in the developing mouse kidney. Our data identifies a subset of binding sites, or ‘regulatory hotspots’ where the engagement of all four factors occurs in close proximity. The putative target genes of their combinatorial action are largely associated with kidney function. Deletion of two of these hotspots for *Six2* and *Wnt4* highlight their roles in target gene regulation and their significance to kidney development. These data suggest that ‘hotspots’ with multi-factor input play significant roles in target gene regulation. Our analysis on the *Br* mutant demonstrated that re-arrangement of the regulatory scenario of *Six2* and *Six3* genes can causes dramatic, predictable effects on their expression and the resulting developmental phenotypes highlighting the importance of appropriate regulatory context to proper gene regulation and biological function.

### Transcriptional hierarchy of nephron progenitors

Our ChIP studies reveal a complex regulatory architecture of the nephron progenitors. Examining co-binding of the four factors suggests each of these genes is itself a target of their combined actions through auto and cross-regulatory inputs, as are a number of other transcriptional regulatory components important for kidney development and nephron progenitor maintenance such as *Sall1* and *Pax2* (S2 Table). By combining our data with insight from previous studies, a hierarchical network starts to emerge. For example, mutational analyses have demonstrated a requirement for the *Hox11* paralogues to activate *Six2* expression in metanephric mesenchyme [12]. Hox11 members complex with Pax2 and Eya1 binding to an enhancer that lies within ~1kb of the *Six2* TSS, in the *Six2-PE* [75]. Hox11 acts as an activator of *Six2* activity and mutations in Hox motifs results in loss of reporter activity in transgenic assays [76]. We have also shown that the Hox motif within the *Six2-DE* is required for reporter activity [28]. Consistent with this data, Hoxd11 is bound at the *Six2-DE* (Fig 2D). However, we did not observe a significant Hoxd11 association to the *Six2-PE* as reported [76]. This discrepancy may result from preferential enhancer usage at different developmental stages. Two previous studies assayed reporter activity of the ~1kb *Six2-PE* at E11.5 [75, 76] while our studies assayed *Six2-DE* activity at E15.5 [28]. Hox11 may be required at the *Six2-PE* to help initiate Six2 expression, but maintenance of expression may then rely, at least partially, with the *Six2-DE* where Hoxd11 is engaged at E15.5. Additionally, Osr1 and Wt1 are enriched at the *Six2-DE* compared to the *PE* (Fig 2D), as is Sall1 [29], supporting multifactor input at the *DE* as an important mechanism of *Six2* regulation. However, Six2 is bound at both the *PE* and *DE*, though *PE* association is weaker (Fig 2D), suggesting both may contribute at some level to the maintenance and autoregulation by Six2 itself. Unfortunately, technical difficulties preclude detailed temporal analysis of engagement in the small numbers of cells that are the foundation of the nephron progenitor pool.

When assessing the targets unique to any transcription factor combination, the greatest enrichment for genes with expression within the nephron progenitors, either in self-renewing or differentiating cells, generally occurred when they were complexed with Six2 (Fig 3D). Hoxd11 showed the lowest levels of enrichment for these targets when engaged with Osr1 or Wt1 in the absence of Six2, suggesting that its primary regulatory functions rely on engaging with Six2. Taken together, these data suggest Six2 acts as a master regulator: co-engagement with Six2 predicts a higher probability of regulatory functions within nephron progenitors.

### Transcriptional factors: Activator, repressor and enabling interactions

Osr1 has been described as a transcriptional repressor in vertebrate kidney development [77]. Xu et al. showed that Osr1 works with the Groucho family members and represses activation of a *Wnt4* enhancer specifically in Six2+ nephron progenitors [17]. Consistent with this result, Osr1 associates with the *Wnt4* enhancer in our ChIP assay (Fig 2D). Additionally, other genes that are not present in the nephron progenitors but rather in differentiating structures such as *Pax8* and *Lhx1* are also bound by Osr1 suggestive of a repressive role (Fig 3C, S2 Table, [49, 56]). However, Osr1 is also bound near genes actively expressed in nephron progenitors such as *Six2* and *Osr1* itself (Fig 3C, S2 Table, [2, 16, 69]). Therefore, our data suggest a more complex relationship than Osr1 simply repressing transcription at all engaged targets. Further, our previous ChIP studies supported dual roles for Six2 in activating transcription within nephron progenitors but also engaging at targets silent in progenitors but activated as progenitors differentiate towards nephrons [3, 28]. Similarly, Hox11 has been characterized as an activator, specifically of *Six2* expression [76]. Consistent with this view, Hoxd11, is bound near nephron progenitor-specific genes but like Six2 binding is also prominent around differentiation targets (Fig 3C, S3 Table). Similarly, these observations extend to Wt1 nephron progenitor targets. Engagement most likely reflects dual activator and repressor actions of these complexes and which activity could be dependent on currently unidentified co-bound factors. Conversely, factor engagement at differentiation-specific gene targets may facilitate or enable subsequent activation of enhancers for differentiation-associated genes following the induction of nephron progenitors. In this scenario, multi-factor engagement may be necessary but not sufficient for target activation for differentiation associated genes. Additional factors or modification of existing transcriptional components following progenitor commitment may modify the action of these regulatory complexes.

### Genomic co-localization of transcription factors in nephron progenitor cells

We observed a highly significant overlap of transcription factor binding in nephron progenitors. Motif analysis of each ChIP dataset showed *de novo*, factor-specific motifs were the most enriched (Fig 1D), supporting direct protein-DNA binding. Additionally, our EMSA assays confirmed factor binding to each motif (S2 Fig). On the other hand, previous studies have shown that Six2 can complex with a number of transcription factors, including Hoxa11 [28] and Osr1 [17] *in vitro*, and Eya1 and Sall1 both *in vitro* and *in vivo* [29, 78, 79]. Osr1 has also been shown to interact with Wt1 *in vitro* [80]. These studies support protein-protein interactions amongst these factors and may account, in part, for the multi-factor co-localization on specific genomic targets. Additionally, our kidney immunoprecipitation data suggests that Six2 can interact with Wt1 and Hoxd11 (Fig 2E), confirming such complexes exist *in vivo*. However, without confirming the co-association of these factors on any genomic loci at the same time and in the same cell, we can only suggest their combined function. The association of each factor with its own DNA target and co-association with each other adds to the difficulty of predicting the actions of the regulatory circuit. Further, it is likely that there are significant components yet to be discovered. For example, all of the ChIP datasets recovered a bHLH motif amongst the most-significantly enriched motifs (S1F Fig). Whereas Myc is a bHLH transcription factor that has been shown to complex with Eya1 and Six2 in the kidney [79], and loss of function *Myc* mutants argue for a role in kidney development [81], the recovered motif is distinct from the conventional Myc-Max target site [44], suggesting a role for another, unidentified family member.

### Target gene functions in nephron progenitors

In addition to identifying target genes with known function during kidney development, we also uncovered novel putative targets of the four factors (see S2 Table for list of all target genes). Bmper is a secreted protein that interacts with Bmp proteins and inhibits their function [82]. Inactivation of *Bmper* in the kidney leads to mild hypoplasia [83]; Bmp signaling plays important roles in the progenitor self-renewal and differentiation [84]. Six2 and the other factors may help fine-tune the level of Bmp signaling through activation of *Bmper*. Rspo1 is a secreted protein that binds to G protein-coupled receptors that activate Wnt signaling and its function has been implicated in multiple developmental systems [85]. Rspo1 could have a role in modulating Wnt signaling in the nephron progenitor niche although *Rspo1* mutants have no obvious kidney phenotype, these mutants have not been analyzed in depth [85]. We also identified other modulators of Wnt signaling within our data. *Shisa2* is reported to attenuate Wnt and Fgf signaling during development [62]. *Shisa2* is expressed in the nephron progenitors along with its related family member *Shisa3* (S2 Table). Other targets like *Tsc22d1* and *Mgat5* are reported to display kidney phenotypes. *Mgat5* is expressed in differentiating structures including podocytes (S2 Table, Eurexpress, www.eurexpress.org, [86]) and shows a glomerular phenotype [51]. *Tsc22d1* is expressed in the nephron progenitors (S2 Table) and mutants have small kidneys [50]. Given the current associations of known targets with kidney development and disease, it is likely that functional analysis of new targets predicted here will identify additional regulators of mammalian kidney development.

From our analyses, the majority of significant targets fall under the control of all four factors. These genes fall into multiple functional categories from transcriptional regulators like *Six2*, *Sall1*, and *Pax2* to signaling factors like *Fgf9* and *Wnt4* to cell cycle regulators such as *Ccnd1* and matrix proteins such as *Lamb1* (S2 Table). This suggests that these transcription factors control many different aspects of progenitor cell biology. Fewer targets with known kidney functions emerge from the interaction maps where one of more the factors was not bound at the putative regulatory region (S5 Table). However, *Eya1*, *Wt1*, and *Bcam* lacked an Osr1 association in combined factor interaction analysis (S6 Table) but are well known for their early roles in the kidney program [18, 21]. *Bcam*, encodes a surface receptor which binds laminin and is expressed at increasing levels in differentiating progenitors (S6 Table). Knockouts display glomerular abnormalities suggesting important functions in the kidney [87]. *Phgdh*, a Six2-independent target with highest expression in nephron progenitors (S6 Table) participates in L-serine synthesis and knockouts are embryonic lethal [88].

### Deletion of regulatory hotspots

Enhancers directing *Six2*-like and *Wnt4*-like reporter gene expression [28], identified as ‘regulatory hotspots’ co-bound by Six2, Hoxd11, Osr1, and Wt1 in the data here, were shown to play roles in regulating activity of both gene targets. Kidney phenotypes were observed in embryos homozygous for the enhancer deletion (*Wnt4-DE*) or when combined with protein null mutations (*Six2-DE* and *Wnt4-DE*). While the study identified functional enhancer regions, neither works alone in regulating normal transcript levels in the target cell type. An alternative proximal enhancer has been documented for *Six2* [76, 89]. This proximal enhancer lies a few hundred base pairs upstream of *Six2’s* transcriptional start site and strongly binds Six2, but not the other regulatory factors analyzed here. Alternative enhancers have not been functionally demonstrated for *Wnt4*. In summary, our studies provide evidence to support a focus on multifactor input to prioritize functional analysis of large datasets emerging from ChIP-seq studies. CRISPR/Cas9 deletion of an enhancer region >100kb from the TSS for Sox2 that is co-bound by multiple transcription factors regulating pluripotency (Oct4, Sox2, Nanog, and Klf4 [90, 91]) provides another example of this strategy to identify strong, *bone-fide* components of the regulatory genome.

### Topological rearrangements in the *Br* mutant and cis regulation of *Six2*

Individual enhancer action depends on the larger context of the chromosomal landscape. Our demonstration that the inversion in *Br* mutant strain, repositions *Six2* and *Six3* in a new regulatory landscape modifying enhancer interactions that likely contribute to altered features of each gene’s regulation. Each gene exists in a distinct TAD that is likely enforced by the action of a CTCF-dependent boundary element between the two genes. In *Br* heterozygous and mutant alleles, *Six3* is ectopically expressed in nephron progenitors: the boundary element no longer separates the *Six3* promoter from the *Six2-DE*. We hypothesize that this enhancer, and potentially undefined regulatory information 5’ to this enhancer, dominate over other regulatory information that might be present within the *Six3* flanking region. As a result, the *Six2-DE* drives *Six3* expression in nephron progenitor cells while *Six3* expression is lost from its normal lens expression domain. Even though Six3 was detected in nephron progenitors in *Br/+* mutants, Six3 is a member of a functionally divergent sub-group of Six factors [92]. Consequently, Six3 activity failed to compensate for loss of Six2 and *Br/Br* mutants resemble *Six2* null mutants [72].

Interestingly, even though there is no alteration in *Six2-PE* position relative the *Six2* gene, the *Six2-PE* is not sufficient to drive levels of *Six2* which maintain nephron progenitor development in the context of the inversion. Thus, if the *Six2-PE* were capable of sustaining normal *Six2* levels, the inversion may prevent *Six2-PE* engagement with regulatory factors necessary for its activation. Alternatively, there may be distinct enhancers other than the *Six2-PE* that are required for *Six2’s* expression. Six2-bound putative regulatory regions lie upstream of *Six2-DE* (S8 Table, Fig 6B) and these would be predicted to disengage from *Six2* regulation in the *Br* inversion.

Topological domains are highly conserved between cell types and across mammalian species [70]. Recent studies have shown that alterations in TADs and CTCF site orientation can affect chromosome architecture and result in altered gene expression [93, 94]. Specifically, several limb malformations in the human were attributed to the rearrangement of TADs and disrupted boundaries. When genetically modeled in the mouse, altered gene expression suggests a mechanism for driving the limb malformations [93]. The type of topological rearrangements described here could play a role in a subset of the congenital anomalies of the kidney and urinary tract (CAKUT) syndrome. Importantly, these micro-rearrangements would not be detected in traditional exome screens. Even whole genome sequencing approaches required tailored alignment algorithms to uncover the junction fragments for the rearrangements as performed here. Together, these studies highlight the importance of non-coding DNA and chromatin architecture to the appropriate regulation of gene expression and the resulting phenotypic consequences incurred by rearranging the regulatory landscape.

### Limitations of the transgenic approach

In addition to Six2, Hoxd11, and Osr1, we attempted to generate transgenic lines for other important regulatory factors including Wt1, Hoxa11, Pax2, Sall1, and Eya1. Our goal was to build an extensive regulatory network for the nephron progenitor population and more precisely identify the targets and combinatorial actions of these major players *in vivo*. However, despite considerable efforts, we were unable to establish correctly expressing founder lines for these factors. The *Six2-DE* is not only active in the kidney but *Six2* is expressed in the developing brain, ear, tendons, and smooth muscle [95] and we observed transgene expression in the brain and ear. Some transgenic lines showed a circling behavior, consistent with inner ear defects, along with insufficient kidney expression and thus were not utilized. The ectopic action of a sub-set of factors in these other sites of *Six2-DE* activity may have resulted in severe defects and subsequent lethality. Alternatively, there could be dominant effects within the kidney itself from elevating levels of that factor in the normal nephron progenitor context, though this seems less likely given the absence of a kidney phenotype in Six2, Hoxd11, or Osr1 transgenic strains, a Six2-BF binding profile that was comparable to the native Six2 protein, and the levels of *Six2-DE* activity.

Despite these technical limitations, we were able to generate a core transcriptional network of four factors important for kidney development. Overlap with additional factors may not add much greater insight; comparison with Sall1 and β-catenin targets reveals many of the same nephron progenitor-specific target genes and a lack of nephron progenitor relevant independent regulation by these factors (S1, S5 and S6 Tables). Therefore, the data presented here is likely to highlight some of the most critical regulatory elements and target genes which modulate nephron progenitor programs.

## Materials and methods

### Mouse strains

All surgical procedures, mouse handling, and husbandry were performed according to guidelines issued by the Institutional Animal Care and Use Committees (IACUC) at the University of Southern California and after approval from the institutional IACUC committee. The transgenic construct utilized by Park et al. [28] to test *Six2-DE* enhancer activity was modified to insert PacI and SwaI sites for cloning downstream of the Hsp68 minimal promoter followed by an IRES-NLS-GFP-BirA cassette. However, the IRES-NLS-GFP-BirA cassette was not included in the generation of the Six2-BF line. Each transcription factor of interest (Six2, Hoxd11, and Osr1) was amplified from E15.5 kidney cDNA with a BioTag-3XFLAG sequence on the 3’ C-terminus and inserted into the transgenic vector using PacI and SwaI sites. Transgenes were purified and injected as previously described [28]. F0 animals were genotyped and transgenic animals bred to confirm germline transmission. Embryonic offspring were also analyzed for correct expression patterns of the transgene in the developing kidney.

Cas9-mediated removal of the *Six2-DE* (chr17:85747271–85749534) was performed by identifying optimal gRNAs flanking the enhancer utilizing the CRISPR Design Tool (crispr.mit.edu; 5’ gRNA: gttaccatctacggtgatgc, chr17: 85747271–85747290; 3’ gRNA: gatatgattctcccgagctt, chr17: 85749515–85749537). The gRNAs were cloned into the pX330-U6-Chimeric_BB-CBh-hSpCas9 plasmid (Addgene) as described (‘One-page Protocol for Cloning Using CRISPR Cas9 Backbone Plasmids’ found at http://www.genome-engineering.org/crispr/; [96]). Pronuclear injection of 2.5ng/μl of each construct into B6SJLF1/J x B6SJLF1/J zygotes (The Jackson Laboratory) with transfer to Swiss Webster (The Jackson Laboratory) pseudopregnant females was performed in house. A similar strategy was used to create the *Wnt4-*∆*DE* mouse (chr4:137216986–137217756). The CRISPR Design Tool (crispr.mit.edu) was utilized to identify optimal gRNAs flanking the enhancer (5’ gRNA: aggctgacaagcgaagttac, chr4:137216986–137217008; 3’ gRNA: atgtcggttgattaataatc, chr4: 137217756–137217778). The following primers were used to generate complexes for *in vitro* transcription of the gRNAs using the MEGAshortscript T7 Transcription Kit (Ambion):

Six2-DE 5’ gRNA F: AATAATACGACTCACTATAAGGCTGACAAGCGAAGTTACGTTTTAGAGCTAGAAATAGC,

Six2-DE 3’ gRNA F: AATAATACGACTCACTATAATGTCGGTTGATTAATAATCGTTTTAGAGCTAGAAATAGC,

Wnt4-DE 5’ gRNA F:

AATAATACGACTCACTATAGAGGCTGACAAGCGAAGTTACGTTTTAGAGCTAGAAATAGC,

Wnt4-DE 3’ gRNA F:

AATAATACGACTCACTATAGATGTCGGTTGATTAATAATCGTTTTAGAGCTAGAAATAGC,

Common R: AAAAGCACCGACTCGGTGCCACTTTTTCAAGTTGATAACGGACTAGCCTTATTTTAACTTGCTATTTCTAGCTCTAAAAC.

Cytoplasmic injection of 100ng of each gRNA and 50ng of Cas9 mRNA (TriLink Biotech) into B6SJLF1/J x B6SJLF1/J zygotes with transfer to Swiss Webster pseudopregnant females was performed in house. To establish lines, F0 animals were born and genotyped to confirm presence of the deletion. One animal carrying a deletion from chr17:85747284–85749542 for the *Six2-DE* and a deletion of chr4:137216991–137217771 for the *Wnt4-DE* (each confirmed by Sanger sequencing of PCR product) was mated to C57BL/6J (The Jackson Laboratories) to establish the line. Genotyping primers:

Six2-DE flank F: GCAGAATGAGATTCTGACAGCCCAG

Six2-DE flank R: CAAGGATGTCTTGTTTGGTCCTTGAGTGAG

Six2-DE internal F: GAGGCCCATAAATAAAGCTGGGACG

Six2-DE internal R: CTCCAGTGACAGATACCACTCTTACTG

Wnt4-DE flank F: AAGCCATGAGGAAAAGAGGGTT

Wnt4-DE flank R: TTCTCAACCCCAAACCCCACC

Wnt4-DE internal F: AGTGTGAGGCACTGTGTAGC

Wnt4-DE internal R: GTGGATGCTGCCTTATGGGT

*Six2TGC*^*tg*^ or *Cited1-nuc-TagRFP-T*^*tg*^ lines utilized for FACS were previously described [69] (http://www.gudmap.org/index.html). *Six2GCE*, *Six2CE*, and *Wnt4GCE* mice were previously described [69]. The *Br* mutant mouse and mapping are previously described [97] and experimental protocols were approved by the University of Hawaii Institutional Animal Care and Use Committee.

### ChIP-seq

Wildtype or transgenic kidneys were utilized for ChIP. *Hoxd11-BF*^*tg/+*^ and *Osr1-BF*^*tg/+*^ kidneys were sorted out prior to ChIP by visualizing GFP+ kidneys. ChIP from E16.5 whole kidneys was carried out as previously described [3]. Nephron progenitor purification by MACS prior to ChIP was carried out is described in Brown et al., 2015 [45].

### 4C-seq

Briefly, E16.5 mice kidney cortex cells were dissociated with collagenase/pancreatin as described in Brown et al., 2015 [45], fixed with 1% formaldehyde for 10 min in room temperature in AutoMACS running buffer. The fixed cells were then processed following published protocols [74] to generate 4C libraries. Dpn II and NlaIII were used in the first and second restriction enzyme digestions, respectively. In order to create a view point from *Six2-DE*, the primers used in 4C-PCR:

Six2DE DpnII reading F: tccctacacgacgctcttccgatctGTTCTGAAAGAGCCGTGTAGGGATC

Six2DE NlaIII noReading R: gtgactggagttcagacgtgtgctcttccgatcGGGGCCCATAAATCGTGATTCAAC

The capitalized letters indicate the complimentary sequences to the genomic view point and the remainder Illumina adaptor sequences. The 4C-libraries were then indexed by PCR and sequenced by NextSeq500. 4C-seq data were analyzed following the workflow provided by 4C-ker [98]. Briefly, the data was mapped to a reduced genome containing 25 bp regions from DpnII sites genome-wide and were subsequently quantified in 3 kb windows to show enrichment. The 4C-seq data is deposited on GEO (GSE90017).

### ChIP-seq data analysis

All ChIP-seq sequences were mapped to the mouse reference genome (mm10) using Novoalign software (Novocraft; parameters: single-end reads trimming 10 bp, polyclonal read filter: 7,10 0.4,2, maximum alignment score acceptable: 120). Mapped ChIP-seq and input data were analyzed using QuEST 2.4 software [99] using a “transcription factor” setting. The false discovery rate (FDR) for detecting the bound regions was evaluated by allocating the same number of mapped reads from a separate mouse input library and performing QuEST analysis using the same parameters. We generated multiple replicates for each ChIP-seq experiment (except Hoxd11 due to technical issues), and used the replicate containing the most peaks using the same peak calling parameters for downstream analyses (see S1G and S3A Figs, G for peak overlap between replicates. The smaller replicates all had >50% overlap with the larger replicate). To account for the innate differences between transcription factors in binding to the genome, we used different parameters in calling peaks of different transcription factors. We used high ratio of peaks with motif and low variability of motif-peak distances as our standard in determining the validity of a data set. See S7 Table for ChIP-seq data information and parameters in peak calling.

In this paper, overlapping sites are defined as those with ChIP-seq peak center distance <150 bp from each other unless otherwise specified. To evaluate the statistical significance of two sets of peaks overlapping each other (Fig 2A), we performed binomial test with the null hypothesis that peaks fall randomly into open chromatin regions in nephron progenitors. To determine the open chromatin regions in nephron progenitor, we performed ATAC-seq [100] in MACS-purified nephron progenitors [45]. We called ATAC-seq peaks with QuEST using the ‘transcription factor’ setting following threshold of fold enrichment > 10. Then we extended the ATAC-seq peak coordinates by 150 bp to both sides, resulting in a total size of accessible chromatin as 21856500 bp. This process is modeled as following:

$$N_{A,B}∼Binom(N_{A},p_{B})$$

$$p_{B}=\frac{{300∙N}_{B}}{{Size}_{accessible\;genome}}$$

where *N*_*A*,*B*_ is the number of peaks in set A that overlap with set B, and *p*_*B*_ is the probability of a randomly located peak overlapping with set B. Information on all ChIP-seq samples presented in the paper can be found in S7 Table. The ChIP-seq and ATAC-seq data is accessible from GEO (GSE90017).

### DNA sequence motif analysis

All de novo motif discovery work was carried out using MEME (Multiple Em for Motif Elicitation, [101]). To find the most enriched motifs for each transcription factor, MEME was run on a pool of 100 bp sequences around the predicted peak center for the top 1000 ChIP-seq peaks called from each data set (or all peaks if number of the total peaks is less than 1000). The locations of motif within 300 bp of peaks are found by FIMO (Find Individual Motif Occurrences, [102]) with data set-specific p-value threshold setting (Six2 motif: 2e-4; Hoxd11 motif: 2e-4; Osr1 motif: 2e-4; Wt1 motif: 2e-5; bHLH motif: 2e-5). We model the appearance of a motif near a set of peaks as following:

$$N_{A,m}∼Binom(N_{A},p_{m})$$

$$p_{m}=\frac{N_{m}}{300∙N_{A}}$$

where *N*_*A*,*m*_ is the number of motif *m* found in +/-150 bp of the peak set *A*; *p*_*m*_ is the probability of finding such motif near a matched random set with the same number of peaks in *A*. Since promoter regions of genes are GC-rich, resulting higher rate of discovering GC-rich motif (Wt1, Osr1 and bHLH) than TA-rich motif (Six2, Hoxd11) in promoter regions. To address this bias, we created the matched random set of peaks by picking genomic coordinates with the same distances to the nearest TSS as the observed peaks set, but with permutated nearest genes. We then screen the matched random peaks for the same motif to obtain *N*_*m*_.

### Genomic Regions Enrichment of Annotations Tool (GREAT) analysis

GREAT GO analysis was performed utilizing the online GREAT program, version 2.0 [43]. Gene regulatory domains utilized for region annotation were defined as minimum 5.0 kb upstream and 1.0 kb downstream of the TSS, and extended up to 500.0 kb to the nearest gene’s minimal regulatory domain (‘single nearest gene’ option). GO Biological Processes annotations were assessed for each peak category.

### Region-based enrichment analysis

To infer the statistical significance of a set of ChIP-seq peaks found near a set of genes (Fig 3), we performed the following analysis. We assigned +/-500 kb from TSS of a gene as its ‘regulatory domain’. We then calculated the probability of a selected set of ChIP-seq peaks falling into the merged regulatory domains of a list of selected genes. This is modeled by a binomial process with the null hypothesis that each peak falls uniformly throughout the genome.

$$N_{A,x}∼Binom(N_{A},p_{x})$$

$$p_{x}=\frac{1}{GenomeSize}\sum_{i\in \{G_{x}\}}\hat{{Size(RR}_{i})}$$

*N*_*A*,*x*_ is the number of peaks in set *A* that fall into the regulatory domains defined by the gene list *G*_*x*_. *p*_*x*_ is the probability of a peak falling into regulatory domains defined by the gene list *G*_*x*_, assuming the peak randomly falling on any position in the genome. *Size*(*RR*_*i*_) is size of a regulatory region after resolving the overlap with any nearby regulatory regions.

We found that each observed set of peaks fall into any random sets of regulatory domains more often than expected, which is not observed when doing the same experiment using random sets of genomic coordinates. To control this background over-representation, we obtained a background enrichment ratio over random sets of regulatory domains by

$$r_{A,x}=\frac{1}{n}\sum_{i=1}^{n}N_{A,{xr}_{i}}/(N_{A}p_{{xr}_{i}})$$

where *r*_*A*,*x*_ is the normalizing factor for a specific set of peaks A. $N_{A,r_{i}}$ is the number of peaks from set A that fall into a the regulatory domain defined by a random list of genes $G_{{xr}_{i}}$ which contains the same number of genes as *G*_*x*_. Therefore, the final binomial model we used in the analysis is

$$N_{A,x}∼Binom(N_{A},p_{x}r_{A,x})$$

### Fluorescence-activated cell sorting

Cortical tissues of E16.5 or P2 kidneys from *Six2TGC*^*tg/+*^ or E16.5 *Cited1-nuc-TagRFP-T*^*tg/+*^ embryos were dissociated as described in Brown et al., 2015 [45]. The dissociated cells were resuspended in autoMACS buffer (Miltenyi Biotec) and passed through a 40 μm nylon filter to obtain single cells. The respective GFP+, GFP-, or RFP+ cells were then isolated with the BD FACSAria II.

### RNA-seq analysis

RNA was isolated from FACS isolated cells using the QIAGEN RNeasy Micro Kit. RNA was submitted to the USC Epigenome Center for library preparation and sequencing on the Illumina HiSeq 2000. All RNA-seq reads were aligned to the mouse reference genome (mm10) using the TopHat2 [103]. Sequences have been deposited in GEO, accession number GSE90017. Quantification of RNA-seq reads to generate RPKM was performed by Partek Genomics Suite software, version 6.6 (St. Louis, MO, USA). TPM was calculated by dividing the RPKM by the mapping ratio of the library to exon regions of the genome. To identify genes differentially expressed in a cell type, we select those with a fold difference > 3, TPM > 5 and p-value < 0.05. Sample information can be found in S7 Table. A complete list of all annotated genes and their coordinating RNA-seq data can be found in S3 and S4 Tables, and coordinating ChIP-seq data can be found in S8 Table. Gene ontology analysis of gene lists was carried out by PANTHER [104]. For the gene set analysis in Fig 3D, we selected the enriched gene lists using the following metrics: nephron progenitor-enriched (TPM > 10 in E16.5 Six2GFP+ cells and fold change > 2 in E16.5 Six2GFP+ vs. Six2GFP- cells), self-renewing nephron progenitor-enriched (TPM > 10 in E16.5 Cited1RFP+ cells and fold change > 2 in E16.5 Cited1RFP+ vs. P2 Six2GFP+ cells) and differentiating nephron progenitor-enriched (TPM > 10 in P2 Six2GFP+ cells and fold change > 2 in P2 Six2GFP+ vs. E16.5 Cited1RFP+ cells). S5 and S6 Tables are pre-filtered to show the nephron progenitor-enriched genes only, but entire lists can be viewed by releasing the filter.

### qPCR

qPCR reaction was performed with Luna Universal qPCR Master Mix Protocol (New England Biolabs) on a Roche LightCycler 96 System. The primers used in this paper includes:

GAPDH F: AGGTCGGTGTGAACGGATTTG

GAPDH R: TGTAGACCATGTAGTTGAGGTCA

Six2 F: CACCTCCACAAGAATGAAAGCG

Six2 R: CTCCGCCTCGATGTAGTGC

Pax2 F: AAGCCCGGAGTGATTGGTG

Pax2 R: CAGGCGAACATAGTCGGGTT

Wnt4 F: AGACGTGCGAGAAACTCAAAG

Wnt4 R: GGAACTGGTATTGGCACTCCT

### *In situ* hybridization

*In situ* hybridizations were performed on frozen sections as previously described (https://www.gudmap.org/Research/Protocols/McMahon.html). The primer sequences used to generate *Wnt4* probe template are:

F: GAGAAACTCAAAGGCCTGATCCA

R: TAATACGACTCACTATAGGGGGCTTTAGATGTCTTGTTGCACG

### Electrophoretic mobility shift assay (EMSA)

EMSA was carried out using Glutathione S-transferase (GST)-tagged recombinant proteins purified from bacterial lysates. To produce the protein, bacterial expression constructs (pDEST15 backbone) were prepared using the Gateway system. BL21-AI One Shot (Life Technology) chemically competent cells were transformed and grown to OD600 = 0.6 before induction with 0.2% L-(+)-arabinose (Sigma) for 3 hrs at 37 °C. The bacteria pellets were resuspended in lysis buffer (20 mM Tris-HCl, 150 mM NaCl, 1% TritonX-100, 1x protease inhibitor, 5 mM DTT) and incubated with 1 mg/mL lysozyme (Sigma). The lysates were sonicated with Branson digital sonifier at 50% amplitude for 90 s. After sonication, supernatant of the lysates were incubated with 5mL glutathione-agarose beads (Sigma) per 1 L bacteria culture for 1 hr at 4°C. The beads were washed with 1% TritonX-100/PBS and eluted with elution buffer (20 mM Tris-HCl, 150 mM NaCl, 15 mg/mL (50 mM) reduced glutathione, 1x protease inhibitor). The eluted protein was concentrated to at least 10 mg/mL using Amicon Centrifugal Filter Unit with the right filter size. The concentrated protein was diluted with PBS and concentrated again to exchange buffer. To perform the EMSA experiments, the recombinant protein was incubated with biotinylated DNA probe for 30 min at room temperature, then the mixture was run through a native TBE gel. The gel was transferred to a nitrocellulose membrane, which was then illuminated using the LightShift Chemiluminescent EMSA Kit (Pierce). The sequences of DNA probes can be found in S2 Fig.

### Immunoprecipitations

Nuclear lysates were prepared from E16.5 whole kidneys using the Nuclear Complex Co-IP Kit (Active Motif). Normal rabbit IgG or Six2 (Proteintech, 11562-1-AP) antibodies were crosslinked with dimethyl pimelimidate to Dynabeads Protein G (Thermo Fisher Scientific) using the Protein A/G SpinTrap Buffer Kit (GE Healthcare). Nuclear extracts were incubated overnight with beads at 4°C. Samples were washed 5x with TBS+0.1% Triton X-100, and proteins subsequently eluted with 0.1M Glycine-HCl, pH 2.9. Samples were run on a 10% SDS-PAGE gel, transferred to nitrocellulose, and subjected to standard Western blotting protocols using Six2 (Proteintech), Hoxd11 (Abcam, ab60715), or Wt1 (Santa Cruz, sc-192) antibodies.

### Immunofluorescence

Kidneys were isolated at the appropriate stage and fixed in 4% PFA for 1 hour. Cryosections were immunostained as previously described [28]. Antibodies used include Six2 (Proteintech, 11562-1-AP), FLAG (Sigma, F1804), Wt1 (Abcam, ab89901), pan-cytokeratin (Sigma, C2931), Pax8 (Abcam, ab13611), Ecad (Sigma, U3254), Six3 (Rockland, 200-201-A26S), and LTL-FITC (Vector Labs, FL-1321). Images were acquired on a Nikon Eclipse 90i epi-fluorescent microscope or Zeiss LSM 780 inverted confocal microscope.

### *Br* sequencing and mapping

Purified genomic DNA from one wildtype and two *Br/Br* animals was sequenced on the Illumina Hi-seq 2000 and mapped to the mm10 genome using Bowtie2. Specific details of the mapping and allele characterization are described further in S1 Supporting Information. Sequences have been deposited in GEO, accession number GSE90017.

## Supporting information

S1 Fig Additional statistics of Six2, Hoxd11, and Osr1 ChIP-seq data. (A) (Top left) Boxplot shows distribution of QuEST scores of Six2-BF peaks overlapping with Six2-ab peaks and the peaks unique to Six2-BF. (Top right) Scatter plot shows correlation of ChIP-seq reads within 150 bp window of Six2-ab and Six2-BF ChIP-seq peaks. (Middle) Enrichment of the Six2 motif in shared versus Six2-ab unique peaks. (Bottom) GREAT gene ontology analysis of shared peaks and peaks unique to the Six2-ab ChIP-seq. (B) The most enriched motif identified from top Six2-FACS or Six2-ab peaks, its enrichment, and distribution. (C) Immunostaining of E15.5 kidneys for Streptavadin 568 on Hoxd11-BF kidneys to highlight the biotinylation of the BioTag component of the BioTagFLAG (BF). (D) Histograms shows distribution of Six2-BF (top), Hoxd11-BF (middle) and Osr1-BF (bottom) peaks’ distance to the nearest TSS. (E) Pie charts show distribution of Six2-BF (top), Hoxd11-BF (middle) and Osr1-BF (bottom) peaks in the genome. (F) Wt1 and bHLH motifs identified from Six2-BF, Hoxd11-BF or Osr1-BF peaks with MEME. Coverage and p-values were calculated with FIMO results. Smoothened histogram indicates distribution of motif-peak distance. (G) Venn diagrams show overlap of peaks identified from Six2-ab and Osr1-BF replicates ChIP-seq data sets.
(EPS)

S2 Fig Validation of ChIP-seq identified binding motifs by EMSA. (A) (1) Weblogo of Six2 motif and probe sequences, with red bases indicating mutation made in the corresponding probes. WT = Wildtype, M = mutant (2) EMSA result shows binding of recombinant GST-tagged Six2 protein (Six2) or GST control (G) to the indicated probes. (3) EMSA result shows effect of the GST or Six2 antibodies on Six2 protein binding to probes. (4) EMSA result shows binding of Six2 to the WT probe in the presence of the indicated competitor probe. (B) (1) Weblogo of Hoxd11 motif and probe sequences, with red bases indicating mutation made in the corresponding probes. (2) EMSA result shows binding of recombinant GST-tagged Hoxd11 protein (Hoxd11) or GST (G) to the indicated probes and effect of antibody on the binding. (3) EMSA result shows effect of competitors on Hoxd11 protein binding to the probe. (4) Weblogo of published PBM Hoxd11 motif. (C) (1) Weblogo of Osr1 motif and probe sequences, with red bases indicating mutation made in the corresponding probes. UP = UniProbe (PBM) motif, O2 = Osr2 motif [S1]. (2) EMSA result shows binding of recombinant GST-tagged Osr1 protein (O) or GST control (G) to the indicated probes. W = water control. (3) EMSA result shows effect of antibody on protein binding to the indicated probe. (4) EMSA result shows effect of competitors on Osr1 binding to the indicated probe. (5) The published Osr1 motif.
(EPS)

S3 Fig ChIP-seq reveals Wt1-mediated regulatory programs in the developing kidney. (A) Venn diagrams show overlap of (left) Wt1-kidney (whole kidney) replicate ChIP-seq peaks, (right) Wt1-NP (nephron progenitor) replicate peaks. (B) From left to right: the number of peaks from Wt1-kidney (top) or Wt1-NP (bottom) ChIP-seq, the most enriched motif identified from the top 1,000 peaks with MEME (using +/- 50 bp window), coverage, p-value, predicted transcription factor (TF) bound, and histogram showing distribution of motif relative to the peak center (Gaussian kernel smoothening was applied to reveal the trend, green curve). (C) Histograms shows distribution of Wt1-NP peaks’ distance to the nearest TSS using both the ‘single nearest gene’ and ‘basal plus extension’ parameters in GREAT. (D) Pie chart shows distribution of Wt1-NP peaks in the genome. (E) Functional annotation of Wt1-NP peaks using GREAT. (F) From left to right: Venn diagram shows overlap of Wt1-kidney and Wt1-NP peaks, Venn diagram shows overlap target genes of Wt1-kidney-unique or shared peaks with Wt1-NP that are associated with the Gene Ontology term ‘nephron development’, selected genes from the indicated part of the diagram. (G) Venn diagram show overlap of the CTCF-NP replicate peaks. (H) Similar as (B), the motif information of the CTCF-NP ChIP-seq dataset.
(EPS)

S4 Fig E18.5 phenotypes of *Six2*^∆*DE/GCE*^ compared to *Six2*^*GCE/GCE*^ mutants. (A) Brightfield images of E18.5 kidneys from *Six2*^*GCE/GCE*^ mutants and compound heterozygous *Six2*^∆*DE/GCE*^ compared to wildtype and single heterozygous littermates. (B) Samples from *Six2*^*GCE/GCE*^ were compared to *Six2*^∆*DE/GCE*^ collected at E18.5 and stained for Wt1, LTL, and cytokeratin (CK).
(EPS)

S5 Fig Localization of the predicted topologically associating domains around Six2 and Six3 and further characterization of the *Br* allele. (A) Hi-C heatmap from Dixon et al. showing the chromatin interactions and predicted topologically associating domains (TADs) surrounding the *Six2* and *Six3* loci, which are boxed in [70]. (B) Genomic view of the inverted region in *Br* allele, with Six2 ChIP-seq, CTCF-NP ChIP-seq, and 4C-seq data tracks. Pink highlighted region is the inverted region. The dashed square indicates predicted TAD boundary from Dixon et al. [70]. Green arrows point to the 4C-seq peak near the *Six2* TSS which is reduced in the *Br/+* kidneys and the 4C-seq peak near the *Six3* TSS which is gained in *Br/+* kidneys. Gray boxes represent the zoomed views of these regions to the right. (C) Schematic of the wildtype (WT) and *Br* alleles with red boxes to indicate primer loci used for PCR. (D) PCR results confirming the appropriate wildtype and mutant *Br* products for each of 3 wildtype and 2 *Br/Br* samples. (-) = No DNA control. (E) Wholemount *in situ* hybridization for *Six3* on E11.5 embryos of the indicated *Br* genotypes. Arrows highlight the lens expression of *Six3* and its loss in the *Br* mutant. (F) Immunostaining for Six2 and Six3 in the E14.5 cranial base of wild type and *Br* mutants. DAPI (nuclei) is shown in red.
(EPS)

S1 Table Regulatory hotspots in nephron progenitors. Genomic coordinates of regulatory hotspots as identified by co-binding of Six2, Hoxd11, Osr1, and Wt1. Coordinates are listed for each transcription factor bound peak.
(XLSX)

S2 Table Core targets and gene expression in the developing kidney. Gene expression of core targets and listing of all target-associated peaks.
(XLSX)

S3 Table Expression of genes in Six2+ and Six2- fractions of the kidney cortex. Results from RNA-seq of E16.5Six2+ (GFP+) vs E16.5Six2- (GFP-) FAC-sorted cells of the kidney cortex.
(XLSX)

S4 Table Expression of genes enriched in E16. 5 Cited1+ and P2 Six2+ cells of the kidney. Results from RNA-seq of uncommitted (E16.5 Cited1+, RFP+) vs differentiating (P2 Six2+, GFP+) FAC-sorted nephron progenitors.
(XLSX)

S5 Table Putative targets of two and three factor unique regulation. Targets, their RNA-seq expression, and associated peaks. Individual sheets are labeled for each two or three factor combination. Combinations of interest were identified from Fig 3D where they showed an enrichment over the whole transcriptome for nephron progenitor specific targets.
(XLSX)

S6 Table Putative targets of single factor unique regulation. Targets, their RNA-seq expression, and associated peaks. Individual sheets are labeled for each unique factor. Targets unique to Six2, or any of the other factors independent of Six2.
(XLSX)

S7 Table Sample information for ChIP-seq and RNA-seq data. (XLSX)

S8 Table All annotated genes and their associated RNA-seq and ChIP-seq data. (XLSX)

S1 Supporting Information Supplemental materials and methods and references. (DOCX)
