# Supplementary material for: Transcriptional regulatory control of mammalian nephron progenitors revealed by multi-factor cistromic analysis and genetic studies
Source: PLoS Genet. 2018 Jan 29;14(1):e1007181. doi: 10.1371/journal.pgen.1007181 (PMC5805373; doi:10.1371/journal.pgen.1007181)
Supplement: S1 Supporting Information — (DOCX) [file pgen.1007181.s014.docx]

## Supplemental Materials and Methods

Our analysis pipeline consists of the following steps: (1) adapter removal, (2) splitting the reads, (3) mapping to genome, (4) extracting sequenced reads of interest, (5) *de novo* assembly, (6) Identifying the breakpoints, and (7) analyzing the reads supporting the candidate breakpoints.

### 1) Adapter removal

In order to remove the adapters from the sequences, first we obtained the adapter sequence, which was the illumina universal adapter: AGATCGGAAGAG. Then we searched for 5’ and 3’ ends of the adapter with all the sequenced reads. In order to call an adapter in the read, we required at least six nucleotides anywhere in the sequence to exactly match the 5’ or 3’ end of the adapter. Furthermore, we required matching at least four following nucleotides with at most one mismatch allowed. After calling the contaminated reads we have removed the adapter nucleotides and any other nucleotide sequenced following the adapter in the read. Table 1 shows the statistics of adapters and the rate of contamination in each data set.

Table 1. Adapter contamination statistics

| **Library** | **Total Sequenced Reads** | **Average length**  **(minus N)** | **Reads with adaptor** | | **Average length (minus adaptor)** |
| --- | --- | --- | --- | --- | --- |
| 6-Br-BR | 218,104,895 | 150.999 | 9,182,646 | 4.21% | 149.2 |
| 7-plus-plus | 225,130,156 | 150.999 | 8,298,704 | 3.69% | 149.1 |
| 9-Br-Br | 201,431,091 | 150.999 | 7,393,954 | 3.67% | 149.5 |

### 2) Splitting the reads

Since our most probable hypothesis is that a segment of genome is inverted due to X-ray radiation in the mutant mouse, a preprocessing step is needed before we proceed to mapping. If we map the sequenced reads before this step, the reads covering the potential breakpoints may either not map at all or map to the wrong location in the genome. These reads in particular have critical information for identifying the location of breakpoint. In order to prevent these reads from being falsely discarded we split all the reads in half. Based on the “pigeonhole principle” if one breakpoint is being covered by a read, then at least one half of the read should map uniquely. By doing this step, we map at least half of the reads that are covering the breakpoints and by recovering the other half that was not mapped due to the covering the breakpoint we can obtain critical information about the breakpoints. Without this step, no assembly would ever assemble a contig spanning through the breakpoints.

### 3) Mapping to the Genome

After splitting the reads in half, at this point for each of the three libraries in Table 1 we have four sequence libraries: two halves of the reads for each of the two ends of the fragments. We mapped all the data sets to the genome (mm10) using the bowtie2 software independently. We used the default set of parameters of bowtie2 for mapping, which allows up to 6% mismatches and retained only reads that could be unambiguously mapped to a single location. Table 2 shows the aggregate mapping statsitic for each library.

Table 2. Mapping statistics

| **Library** | **Total Sequenced Reads** | **Mapped reads** | | **Average length** | **Average fragment length** | **Standard dev. Fragment length** | **Average mismatches** |
| --- | --- | --- | --- | --- | --- | --- | --- |
| 6-Br-BR | 218,104,895 | 184,942,945 | 84.8% | 149.0 | 285.0 | 93.2259 | 5.91 |
| 7-plus-plus | 225,130,156 | 190,017,499 | 84.4% | 149.3 | 287.7 | 93.8093 | 5.99 |
| 9-Br-Br | 201,431,091 | 171,112,921 | 84.9% | 149.3 | 284.9 | 93.6014 | 6.19 |

### 4) Extracting sequenced reads of interest

At this step, we extracted all the reads that at least one part of them was mapped to the region of 1Mb around the six2 gene. We brought back the mates of the reads as they might have spanned through the breakpoints. This set of reads corresponds to all the reads that cover the region around the six2 gene including the reads that are covering the potential breakpoints.

### *5) De novo* assembly

At this step, we assembled the extracted reads to obtain a set of contigs for each library. We used the velvet program to perform the assembly in paired end mode. We used velvet optimizer to optimize the parameters of assembly to find the best set of contigs out of the reads, as follows:

> VelvetOptimiser.pl -t 4 -s 20 -e 100 -f '-shortPaired -separate -fastq 6-Br-Br_S13_1.fastq 6-Br-Br_S13_2.fastq'

The optimum length of the overlap for these reads was reported as 75 nucleotides. Table 3, presents the number of reads used for assembly, the number of contributing reads and the number of resulting contigs for each library.

Table 3. Contig statistics

| **Library** | **Total number of reads** | **Total number of contributing reads** | **Number of contigs** |
| --- | --- | --- | --- |
| 6-Br-Br | 70,932 | 29,714 | 1,370 |
| 7-plus-plus | 70,987 | 30,022 | 1,493 |
| 9-Br-Br | 60,754 | 26,175 | 1,402 |

**6) Identifying breakpoints**

At this step we proceed to analysis of the resulting contigs. We first used BLAT, to find the matches for these regions. Then we calculated the percent-identity scores in BLAT results and the fraction of the contigs that was mapped to the reference genome. In order to find the breakpoints, we applied different filters to remove the contigs that are irrelevant, so we can find the precise location of the breakpoints. The filters are as follows:

- Filter #1:
  - We removed all the contigs that were mapped to reference completely.
- Filter #2
  - We kept the contigs that were mapped to the genome partially, (with less than 50% of the contig being maped), but in that region it was mapped with a high percent identity (>90%). We also kept only the contigs that had any region of non-matching nucleotides only on one end, leaving only less than 10 nucleotides gap in the other end.
- Filter #3
  - We kept the best match for each contig.

Now with these contigs we extract the sequence of the part of them that was not mapped. We mapped these regions to the genome using BLAT as well, but this time we kept the ones that mapped with high percent identity and allowing small gaps on either end of them (<10 nucleotides).

- Filter #4:
  - We kept only the contigs that two parts of them were mapped to different strands.

Table 4. The number of reads after applying each filter

| **Library** | **Mapped on chromosome 17** | **Non-perfect matching**  **(Filter #1)** | **Half mapped well**  **(Filter #2)** | **Best matched**  **(Filter #3)** | **Two parts in opposite strands**  **(Filter #4)** |
| --- | --- | --- | --- | --- | --- |
| 6-Br-Br | 2,599 | 1,717 | 215 | 49 | 26 |
| 7-plus-plus | 2,625 | 2,625 | 390 | 86 | 38 |
| 9-Br-Br | 2,488 | 2,488 | 288 | 70 | 25 |

Up to this point we had a set of candidate contigs that point to the breakpoints, we investigated all of them to see if we can discover the inversion.

After this step we investigated each of the resulting contigs separately, and we were able to find the breakpoints. Figure 1. (A) Shows four contigs breaking at the first break point (2 per replicate). As shown in the figure, three contigs are mapped to the positive strand, and one to the negative strand. An occurrence of a deletion is also obvious in the genome browser tracks. It is worth mentioning that we also see peaks of discordant reads mapping in either sides of the breakpoint. Figure 1(B) illustrates the above-mentioned contigs breaking at the second break point. As shown in the figure, the parts of the contigs going through the first point are mapped to the different strand. Both replicates are consistent.

**7) Computational validation of inversion**

Then we proceed to analyse the reads that are supporting each candidate breakpoint to make sure the breakpoints are due to an inversion event. To do this, first we split the reads to three parts, and did this for both mates for each fragment (because of fragment length and overlapping mates, both mates could potentially contain a breakpoint). So we have 3 parts per end, and 6 per fragment, given that we have 150nt read ends. We mapped these parts to Six2 domain and removed all fragments for which the “parts” are all mapping with concordant strand. For all pairs of same-fragment parts, we computed distance between mapping locations (ignore strand) and we built the histogram of distances. As shown in figure 2 we have a secondary peak in roughly about 325kb distance. This result is consistent with the break point illustrated in the first figure.

Then we took the reads that were used to make one side of these contigs and looked to see where are their mates mapping. For this procedure we used three parts of each read that we discussed in previous figure. As shown in the figure the mates of the reads that are making one contig in one side of the break point are mapping to the other side of the breakpoint. This clearly verifies the inversion.

Figure 1

Figure 2

Figure 3

**Supplemental References**

[S1] Kawai S, Yamauchi M, Wakisaka S, Ooshima T, Amano, A. Zinc finger transcription factor Odd skipped related 2 is one of the regulators in osteoblast proliferation and bone formation. J Bone Miner Res. 2007; 22: 1362-1372.
